# Holocene surface-rupturing paleo-earthquakes along the Kachchh Mainland Fault: shaping the seismic landscape of Kachchh, Western India

**DOI:** 10.1038/s41598-024-62086-z

**Published:** 2024-05-21

**Authors:** Javed N. Malik, Eshaan Srivastava, Mahendrasinh S. Gadhavi, Franz Livio, Nayan Sharma, Shreya Arora, Nicolò Parrino, Pierfrancesco Burrato, Attilio Sulli

**Affiliations:** 1 Active Tectonics and Paleoseismology Laboratory, Department of Earth Sciences, Indian Institute of Technology Kanpur, Kanpur, Uttar Pradesh 208016 India; 2 Dipartimento di Scienze della Terra e del Mare, Via Archirafi 22, Università degli Studi di Palermo, Palermo, Italy; 3 Civil Engineering Department, L. D. College of Engineering, Ahmedabad, Gujarat 380015 India; 4 Dipartimento Di Scienza Ed Alta Tecnologia, Università Degli Studi Dell’Insubria, Via Valleggio, 11, 22100 Como, Italy; 5 Department of Earth and Climate Sciences, Bates College, Lewiston, ME USA; 6 Istituto Nazionale Di Geofisica E Vulcanologia, Rome, Italy

**Keywords:** Natural hazards, Geomorphology, Tectonics

## Abstract

This study explores the seismotectonics of Kachchh in western India, a region with a low-to-moderate strain rate and a history of significant earthquakes, notably the 1819, Mw 7.8 Allah Bund, and the 2001, Mw 7.6 Bhuj. Despite its substantial seismic risk, comprehensive studies on Kachchh’s seismogenic sources are scarce. This is attributed to the concealed nature of active structures, hindering definitive age constraints in paleoseismological research. Our research comprises a detailed paleoseismic analysis of the north-verging, reverse Jhura Fault underlying the Jhura anticline, a segment of the Kachchh Mainland Fault. This fault segment shows evidence of surface-rupturing earthquakes in the area south of the Great Rann of Kachchh. The investigation reveals three paleoseismic events: Event I before 9.72 ka B.P., Event II between 8.63–8.20 ka B.P., and Event III between 6.20–6.09 ka B.P. The elapsed time since the last event on this fault is > 8000 years, suggesting that the area is exposed to a significant earthquake hazard. This highlights the need for more precise characterization of individual seismogenic sources for future earthquake preparedness.

## Introduction

**Figure 1:**
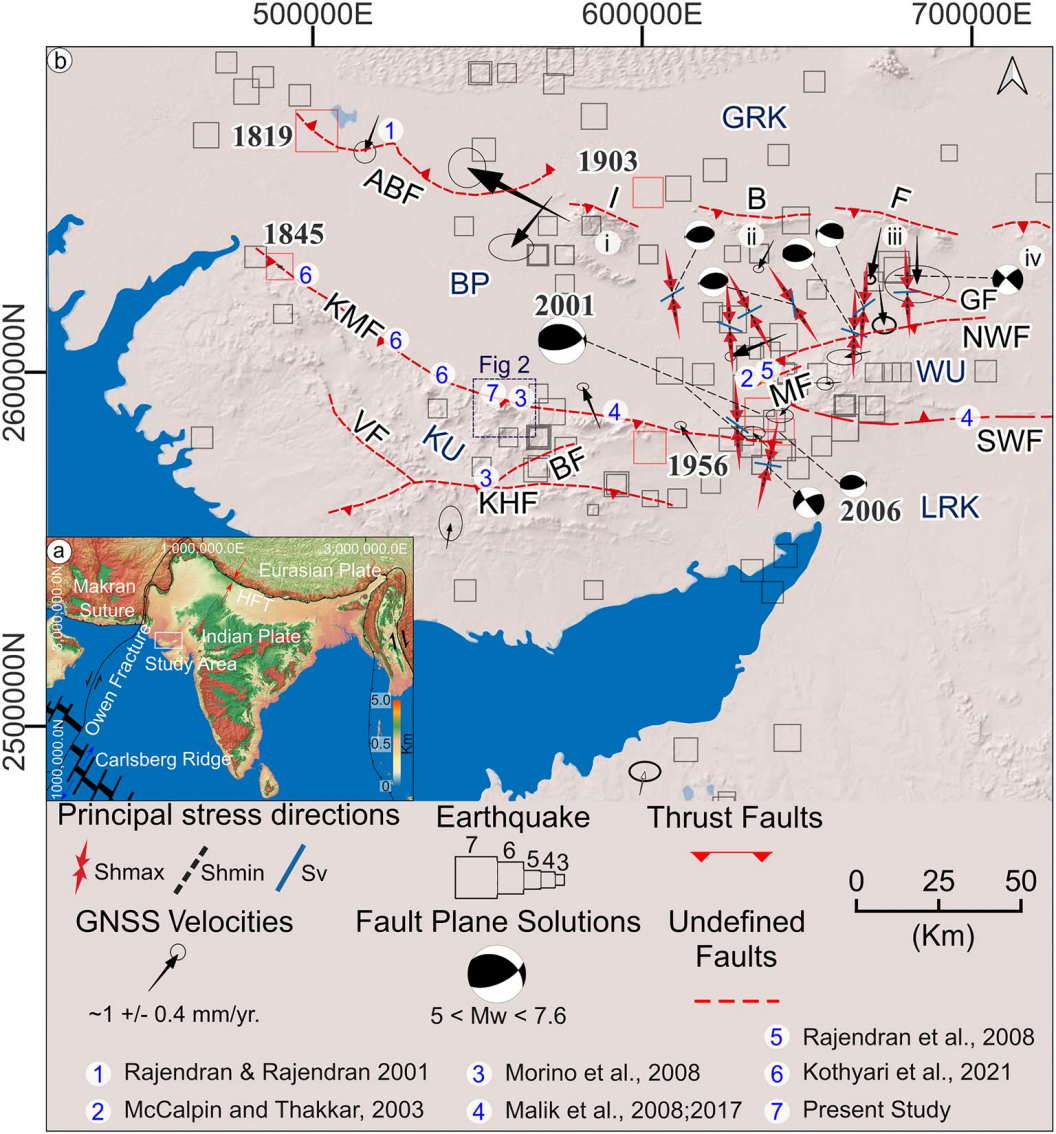
(**a**) Simplified map depicting major structures around the Indian subcontinent, such as the Carlsberg Ridge, Owen Fracture, Makran Subduction Zone, and Himalayan Collision Zone, as well as the study area about these structures; (**b**) seismotectonic-structural map of the Kachchh region. This map categorizes different geomorphic zones as follows: KU (Kachchh Upland), LRK (Little Rann of Kachchh), WU (Wagad Upland), BP (Banni Plain), island belt region (i: Pachchham, ii: Khadir, iii: Bela and iv: Chorar) and GRK (Great Rann of Kachchh). It also identifies key faults, arranged from south to north, including: KHF (Katrol Hill Fault), BF (Bhuj Fault), VF (Vigodi fault), KMF (Kachchh Mainland Fault), SWF (South Wagad Fault), MF (Manfara Fault), NWF (North Wagad Fault), GF (Gedi Fault), IBF (Island Belt Fault), and ABF (Allah Bund Fault). The distribution of seismic activity throughout the Kachchh region, as indicated by documented earthquakes are seen aligned along these major faults. The historical and recent seismic events depicted with red squares—1819 Allah Bund, 1845 Lakhpat, 1956 Anjar, and 2001 Bhuj—alongside the locations of paleoseismic trenches from previous studies. It incorporates principal stress directions, as sourced from the World Stress Map project, GNSS velocities (referenced to ITRF 2008), and fault plane solutions reported by the United States Geological Survey (USGS: https://earthquake.usgs.gov/earthquakes/search). Figure generated with QGIS v. 3.28.11 (https://www.qgis.org/en/site/).

The 2001 Bhuj earthquake (Mw 7.6), which caused about 20,000 causalities, more than 150,000 homeless people, and USD 42.55 million in economic loss, increased attention to the anomalous seismicity of the Kachchh peninsula (NW India). This led to the surge of studies to decipher the seismotectonic framework of this area that also experienced the 1819 Allah Bund (Mw 7.8) and the 1956 Anjar (Mw 6.0) earthquakes (Fig. 1)^1^.

Most active tectonics and paleoseismological research in this low-to-moderate strain rate intraplate region was focused on the 80–90 km long primary surface rupture and widespread seismically induced liquefaction features induced by the 1819 event^2–10^. Moreover, studies following the 2001 event focused on identifying its causative fault using aftershocks relocation, remote sensing, gravity data, and geodetic monitoring^11–22^. These studies reported an E-W striking, south-dipping thrust fault with a coseismic slip of up to 10 m, confined between 10 and 30 km of depth^11–23^. Further, studies on the coseismic surficial deformation of 2001 earthquake, reported probable occurrence of a 0.8 km long primary fault scarp with a dextral component on the Manfara Fault (MF), along with widespread liquefaction and lateral spreading features^24–26^.

The two major Kachchh events (1819 and 2001) had a similar magnitude and secondary deformation, but strikingly different surface rupture lengths^9,10,27–34^. Such difference in surface rupture length between earthquakes that occurred to the North and the South of the Great Rann poses a significant gap in the understanding of the seismotectonic framework of Kachchh and raises an important question, whether the active faulting may occur along structures with different geometrical characteristics? To address this gap, attempts were made in the form of active fault mapping and paleoseismological trenching along Kachchh Mainland Fault (KMF), Katrol Hill Fault (KHF) and South Wagad Fault (SWF) (Fig. 1b).

The first active fault map was published in 2001, which reported active fault traces along the KMF^28^. Later, the only study from the SWF by Malik et al. (2017)^35^, reported a well-preserved deformation zone on a north-dipping thrust fault with three paleoearthquakes. However, the remaining active faults and paleoseismic studies lacked in different ways, especially because evidence of paleo-earthquakes were reported based on liquefaction features, which are secondary evidence related to seismic shaking and may not be very useful for identifying causative faults in the complex tectonic setting of the Kachchh basin^9,10,33^. Further, a series of trenches excavated across the KMF and KHF revealed surface rupturing earthquakes on the south-dipping fault during the Late Pleistocene, but without age constraints^29,31,32^. Kothyari et al. (2021)^34^ explored the western segment of the KMF and unearthed six paleo-earthquakes from six trenches. These findings raised questions about the wide-depositional *hiatus* retrieved in those trenches (i.e., Mesozoic sandstone directly overlain by 10 ka old calcareous sands), and questions the rheology of the layers for arresting such vertical faults. For example, the tendency of a fault to propagate through a medium, with a slip, and the influence of near-surface geology on such behaviour is a primary parameter that can potentially obscure the evidence of surface faulting both in geomorphology and in exploratory trenches^36,37^.

**Figure 2:**
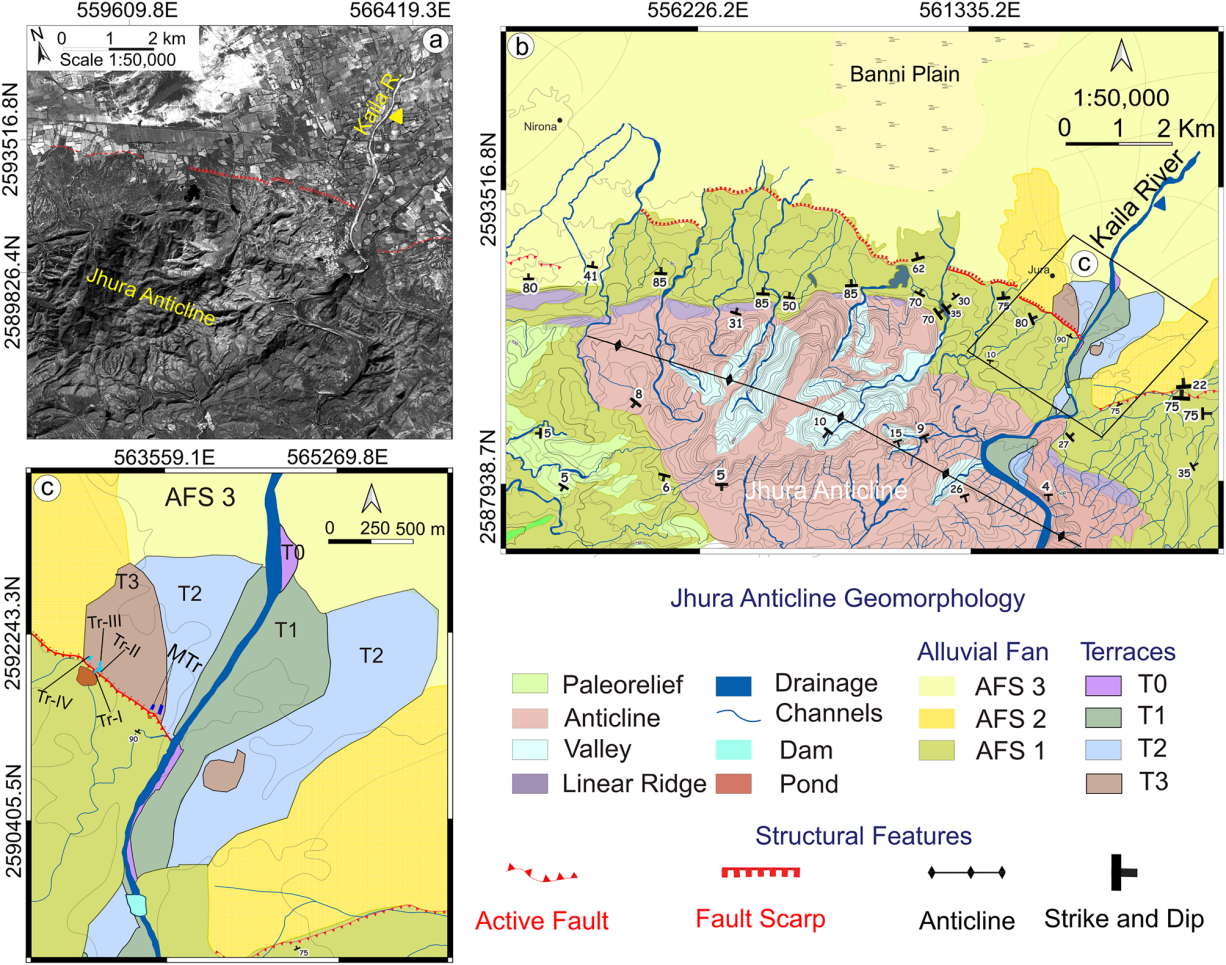
(**a**) Raw imagery of CARTOSAT-I aft (after stereo pair image) dataset^38,39^ of the Jhura anticline and the adjoining region; (**b**) detailed geomorphologic map of the Jhura anticline and the adjoining region; (**c**) detail on the study area: the location of the excavated trenches is shown with Tr-I to Tr-IV along with trench locations (MTr) from Morino et al., (2008). Figure generated with ENVI Classic (https://www.l3harrisgeospatial.com/Software-Technology/ENVI) and QGIS v. 3.28.11 (https://www.qgis.org/en/site/).

Based on the above-mentioned observations, there still remain the following open questions: (a) Can large surface-rupturing events, comparable to the 1819 Allah Bund earthquake, also occur south of the Great Rann? (b) Can these events be constrained using paleoseismology in the late Holocene or Pleistocene and even beyond? (c) Can paleoseismology confirm that Kachchh is among the most active intraplate region worldwide? To address these issues our study involves paleoseismic and morphotectonic analyses on the Jhura Fault (JF), a south-dipping branch of the Kachchh Mainland Fault (KMF) that demarcates the northern boundary of the Jhura Anticline (JA), extending laterally for 15–20 km. This section of the KMF exhibits north-facing active fault scarps that separates older and younger alluvium (Fig. 2). We utilised satellite imagery, photogrammetry, and field data to map the active fault trace, and identified four trenching sites for paleoseismic investigation, dating three paleo-ruptures. Through trishear kinematic inverse modelling, we assessed the fault and fold growth, comparing recent movements to the overall displacement. The present study along JF highlights surface rupturing events, updates the seismic event catalogue, and evaluates earthquake recurrence of the Kachchh region in a global intraplate context.

## Geologic and tectonic setting

The Kachchh intracratonic rift basin formed during the breakup of Gondwanaland in the Late Triassic and lies in the western part of the Indian plate. Its geological evolution, encompassing the breakup, drifting, and collision of the Indian Plate with the Eurasian Plate, involves three key tectonic events^40–44^,: (i) a Late Triassic rifting phase^40^; (ii) an Early Jurassic to Late Cretaceous divergent phase^40,45,46^; and (iii) a post-rifting transpressional phase since the Tertiary ^40,46,47^. The basin is divided into distinct geomorphic zones, including a coastal zone, the central rocky uplands (Kachchh and Wagad Uplands), the Banni-Plain, the Island belt (Pachchham, Khadir, Bela and Chorar), and the saline-waste lands of the Great and Little Rann ^48^ (Fig. 1).

GNSS velocity data reveals an NW–SE oriented shortening in the Kachchh area^49^ of ~ 3 mm/year, with the principal horizontal stress (SHmax)^50^ direction varying from NW–SE to NNE-SSW. The main active structures of Kachchh are the KMF, the SWF, and the ABF bordering the Great Rann to the south and to the north, respectively, which, according to Gahalaut et al. (2019)^49^, should be characterized by a dip-slip rate of ~ 4–6 mm per year (Fig. 1)^49^.

Historically, the 1819 Allah Bund earthquake was the most significant event, creating a 80–90 km long fault scarp with variable height of 4–6 m^4,5^. Its magnitude, estimated to be between Mw 7.6 and Mw 8.2, is debated due to limited data on uplift and subsidence^3,5,8,35,48,49^. The 1956 Anjar earthquake (Mw 6.1), originated near the JA, probably along a segment of the KMF, at the northern boundary of the Kachchh upland (Fig. 1)^3,5,8,51,53^.

Instrumental seismicity in the region has been notably characterized by the earthquake swarms around the Bhuj epicentral area since the major 2001 event^35^. Seismological data from 2000 to 2020 includes several earthquakes with magnitudes ranging from Mw 4 to the main shock of Mw 7.6, with focal depths ranging between 10 and 40 km, primarily near the KMF and SWF ^46,54,55^.

**Figure 3:**
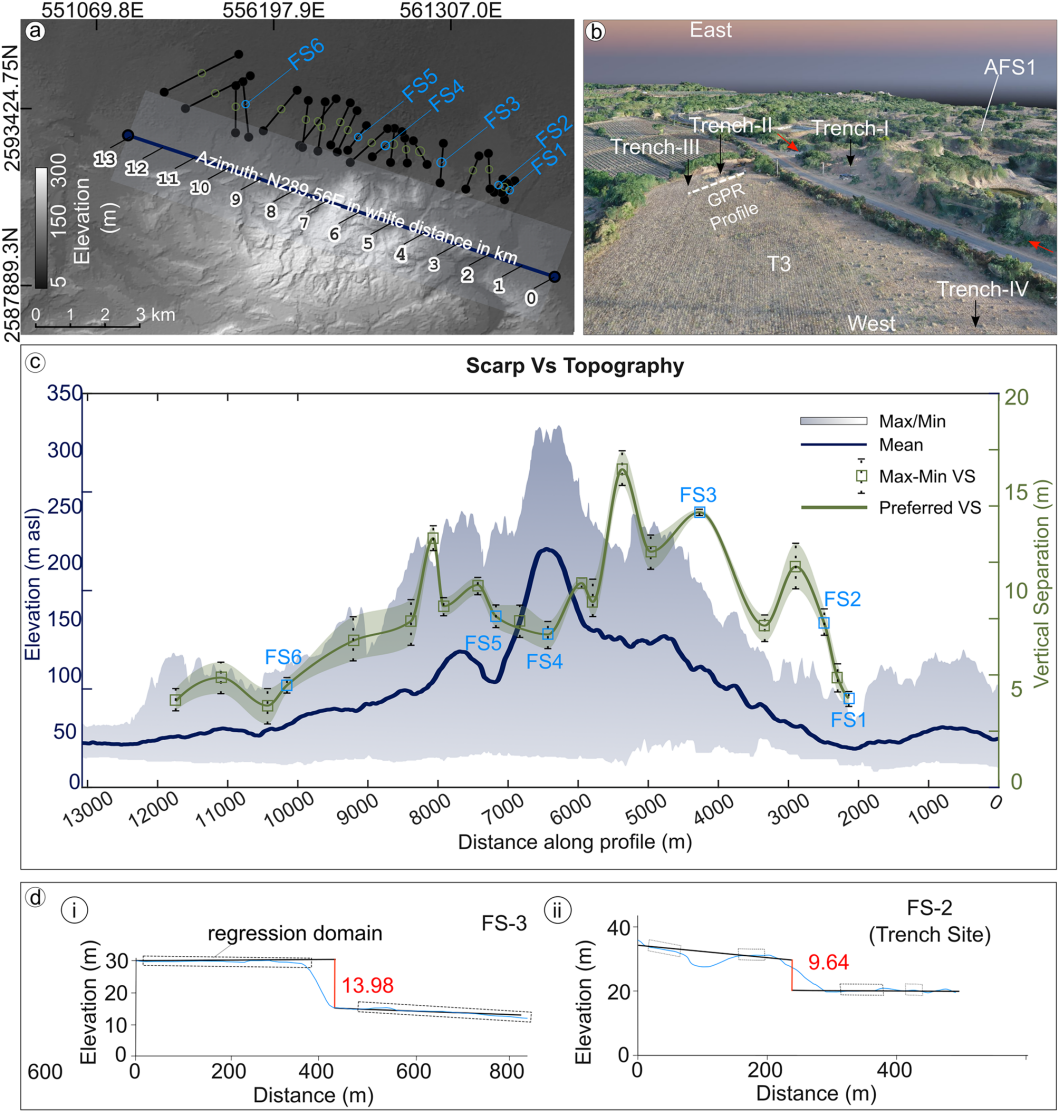
(**a**) CARTOSAT-I Digital Surface Model (DSM) showing swath bounds and the traces of the topographic profiles crossing the fault scarp at the anticlinal front, used for the computation of the vertical separation; (**b**) Slanted UAV imagery exhibiting a locations of the trenches, Ground Penetrating Radar (GPR) profile and high-resolution geomorphic features as depicted in the CARTOSAT-I dataset; (**c**) composite profiles of the Jhura Anticline, vertical separation (VS) is indicated by green, with preferred values marked in square boxes and bounds indicating the standard mean error of the topographic profile of the fault scarp (FS), alongside a swath profile showing the swath bounds as depicted in (**a**), and (**d**) topographic profiles across the Jhura Fault scarp, measured using a 3 m resolution CARTOSAT-I DSM, the trace of which is shown in Fig. 3a, additional topographic profiles are in the Supplementary Data Fig. S2. Figure generated with ENVI Classic (https://www.l3harrisgeospatial.com/Software-Technology/ENVI), Pix4D mapper (https://www.pix4d.com/), MATLAB v.R2023a (https://www.mathworks.com/products/new_products/release2023a.html/), QGIS v. 3.28.11 (https://www.qgis.org/en/site/), and Inkscape v. 1.3.0 (https://inkscape.org/).

The JA lies in the middle of the KMF at an elevation of 300 m above m.s.l. and with a core characterized by the outcrop of Mesozoic rocks, with Tertiary and Quaternary successions covering the anticline limb to the north (Figs. 2, 3; and refer main map Srivastava et al., 2023 for lithological contacts^44^). Deeply incised V-shaped valleys, tight meanders, strath terraces and a prominent E-W to WNW-ESE ranging fault scarp highlights a significant tectonic forcing in its recent landscape evolution^44,52–55^. The fault scarp displaces recent alluvial fans and river terraces across the Kaila River (Figs. 2 & 3). Additionally, it cuts the Jurassic sandstones that dip steeply at 70–80° to the southwest, and sub-vertically dipping Tertiary beds (Figs. S1a,b).

Recent studies on this structure, propose that it is underlined by a 15°, south-dipping thrust fault, that was characterized during the last event by more than 5 m of slip accommodated by drag folding in young alluvium at shallow depths^31^.

## Data and methods

### Dataset gathering and computation

The geological and geomorphological information involving the structural data, geomorphic landforms and the diverse lithological outcrops was obtained through remotely sensed datasets (CARTOSAT-I, 2005–2006: Indian government web portal, https://bhuvan.nrsc.gov.in/home/index.php), official maps (Fig. 1)^9,29–31,33,34,45,46,48^ and field surveys. Data related to historical-instrumental earthquakes were gathered from multiple sources such as the USGS (https://earthquake.usgs.gov/earthquakes/search/), the International Seismological Center (ISC; http://www.isc.ac.uk/iscbulletin/search/catalogue/), and from the Institute of Seismological Research (ISR), Gandhinagar catalogues^30^. The focal mechanisms in Fig. 1 are from the Global CMT Catalogue (https://www.globalcmt.org/). The stress indicators consisting of horizontal maximum (Sh_max_), minimum (Sh_min_), and vertical (S_v_) stress directions are from the World Stress Map (WSM) project (https://www.world-stress-map.org/; Fig. 1)^50,57^. Lastly, the GPS velocities in Fig. 1 are from Gahalaut et al., (2019)^49^.

The declassified CARTOSAT-I panchromatic satellite imagery with stereo vision capability, obtained from the National Remote Sensing Centre (NRSC) (https://www.isro.gov.in/Spacecraft/cartosat-1), has a resolution of 2.5 m and the data was acquired during the 2005–2006 mission. The CARTOSAT-1 satellite acquire two images, one with forward looking (Fore) camera, and another by after (Aft), which are at tilt angles of + 26° and − 5°, respectively. Each imagery set comes with its respective predetermined Rational Polynomial Coefficients, based on their unique orbit and attitude models. These coefficients facilitate the acquisition and metadata necessary for correcting geometric distortions in the imagery^38,39^*.* Using these images and following the methodology of Evans et al., 2008^57^ we employed the ENVI 4.7 software, for generating a Digital Surface Model (DSM) and a 3D Anaglyph (3DA), both characterized by a geometric resolution of roughly 3 m.

The study area was also surveyed using a UAV DJI Phantom 4 pro. It is a quadcopter equipped with an internal sensor of ½0.3″ CMOS, FOV 94° 20 mm (35 mm format equivalent) f/2.8 focus at ∞ lens and operated by a remote control boarded with a mobile device, which has the respective commercial application for mapping such as Pix4D mapper. This survey allowed to produce Hi-Res photograph mosaic for data visualization and geomorphic interpretation.

We started field survey investigations, acquiring multiple ground-penetrating radar profiles. Following the common midpoint approach, the electromagnetic waves provided indications of tectonic perturbations in the subsurface^58^. Optical Stimulated Luminescence (OSL) samples were collected using stainless steel tubes hammered into a clean vertical sections for sample locations. Thereafter, samples were processed at the luminescence facility at IIT Kanpur equipped with the Frantz magnetic separator, the Risø TL/OSL reader and the hyper-pure germanium detectors (HPGe) gamma-ray spectrometer^59^.

### Geomorphic mapping and topographic analyses

Using 3DA and the Hi-Res photograph we mapped the main morphotectonic feature of the study area. Furthermore, we validated the produced thematic maps through field surveys conducted in the time frame starting from January-2015 to July-2018.

Topographic analyses were performed using the terrain profile plugin in QGIS and the MATLAB. Employing this DSM as input data, we used a script to calculate the Vertical Separation (VS) between the hanging wall and footwall block surfaces along the JF trace, following the workflow proposed by DuRoss et al. (2019)^60^. To be sure that we took into account the tectonic forcing in the topographic signal we extracted twenty-two topographic profiles across the mapped Jhura Fault scarp. To do this we also considered the previously detected geomorphic feature with the best preservation and the sparsest vegetation detected also in the Hi-Res photographs and 3DA. Finally, to correlate the long-term deformation with the short-term one we also extracted a topographic swath profile along the strike of the whole JA using the TopoToolbox library^61^.

### Trenching and optical stimulated luminescence dating

**Figure 4:**
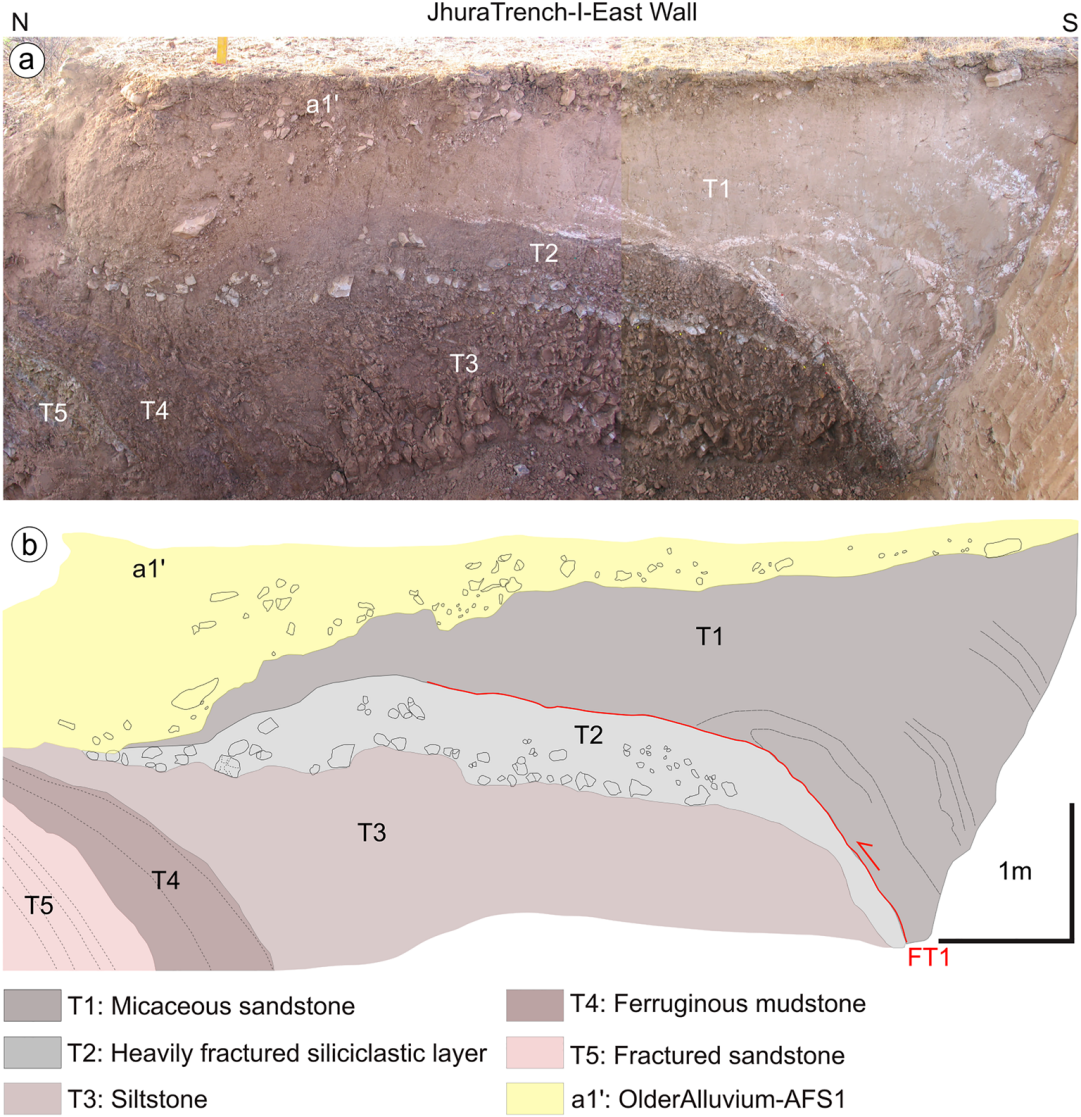
(**a**) A view of the east wall of the Trench-I; (**b**) log of the trench: for a full description, see details in the text.

The paleoseismological trenching was conducted during October–November 2016 and 2017 to identify the signatures paleo-earthquakes along this segment of KMF. The trenching sites were selected considering the scarp profiling, the exposed zone of deformation demarcated by the vertically stacked Tertiary and Mesozoic succession, suggesting propagation of active faulting further north, and, in addition, the high-resolution, subsurface information derived from the GPR profiles (Figs. 3 and 4, and Supplementary Data Figs. S1a-e and S3). We excavated four trenches: one at the base of the fault scarp and the other three on top of a gently sloping surface. Following this, we prepared a trench log for each excavation, which included a description of the lithology, grain size, colour, bedding, and tectonic structures. Photo-mosaics of the trench walls were also prepared and overlaid on the trench log with the embedded information for improved visualization.

For constraining the age model of the sedimentary sequence, we collected twenty-three samples from the exposed succession of both the trench walls and used the OSL dating technique. Quartz grains (90–150 μm) were selected under controlled laboratory conditions. The extraction involved chemical treatment with 1N HCl and 30% concentrated H_2_O_2_ that helped remove carbonates and organic matter. Thereafter chemically treated sediments were magnetically separated from heavy minerals and feldspar and isolate quartz grains. HF treatment was done to remove any remanent feldspar, and then the sample was rinsed with HCl to remove any HF acid-induced fluorite precipitate from the isolated quartz^62–64^. To determine the equivalent dosage (De) of these extracted quartz samples, the traditional single-aliquot regenerative dose (SAR) methodology was used^63,65^.

Following the preparation, eight out of the twenty-three samples, were not further considered for dating. Samples were processed only if they showed a bright quartz signal and a fast component-dominated decay shine-down curve, along with if the recycling and recuperation ratio values deviated by less than 10% and 5%, respectively, from unity^63^. After the De estimation, the dose rates were measured for all samples and was tuned to account for moisture attenuation as obtained in the laboratory. When normalized for field saturation, the water content of a dehydrated and saturated material is referred to as the lifetime water content at burial time (Table 1). Furthermore, the quantification of the impact of cosmic rays on the ultimate dose rate was determined following the methodology established by Prescott and Hutton (1995)^66^.

**Table 1:** OSL ages obtained from the walls of the excavated trenches at the piedmont zone of Jhura anticline. (a) OSL ages from the east wall of trench-II Trench, (b) OSL ages from the west wall of trench-II Trench.

a) OSL ages from the east wall of Jhura Trench												
S. No.	Sample Site	Sample No.*	Unit	Depth (m)	Water Saturation	U-238 (ppm)	Th-232 (ppm)	K count (%)	ED (Gy)	Dose Rate (Gy/Ka)	Age (BP)	Age Model
1	Jhura	JUTLEC 1	d3	1.1	0.668 ± 0.0667	3.66 ± 0.84	16.49 ± 2.57	1.21 ± 0.018	22.64 ± 0.12	3.308 ± 0.192	6840 ± 399	CAM
2	Jhura	JUTLEC 3	d3	2.1	0.53 ± 0.053	3.817 ± 0.490	19.58 ± 0.0192	3.345 ± 0.0238	32.459 ± 0.162	4.112 ± 0.187	7890 ± 362	MAM
3	Jhura	JUTLEC 4	e2	0.64	0.7260 ± 0.0726	1.46 ± 0.009	9.287 ± 0.25	0.34 ± 0.0016	9.025 ± 0.015	1.48 ± 0.034	6099 ± 140	MAM
4	Jhura	JUTLEC 5	d4	1.02	0.53 ± 0.053	1.82 ± 0.011	9.23 ± 0.93	0.9 ± 0.19	13.46 ± 0.24	2.09 ± 0.034	6420 ± 153	MAM
5	Jhura	JUTLEC 6	d3	1.16	0.5096 ± 0.051	2.472 ± 0.097	13.25 ± 124	1.19 ± 0.0127	19.355 ± 0.32	2.801 ± 0.071	6909 ± 210	MAM
6	Jhura	JUTLEC 7	d3	1.58	1.0029 ± 0.1003	2.39 ± 0.028	13.81 ± 1.18	1.22 ± 0.0179	20.085 ± 0.74	2.82 ± 0.067	7110 ± 311	MAM
7	Jhura	JUTLEC 9	c4	2.28	0.8630 ± 0.08	4.17 ± 0.43	23.127 ± 3.28	1.62 ± 0.047	36.60 ± 2.19	4.24 ± 0.192	8632 ± 643	MAM
8	Jhura	JUTLEC 10	c3	1.62	0.9540 ± 0.19	3.92 ± 0.512	23.58 ± 3.47	1.77 ± 2.04	40.45 ± 3.28	4.368 ± 0.201	9261 ± 864	MAM

(b) OSL ages from the west wall of Jhura Trench												
1	Jhura	JFTL1	e2	0.34	0.65 ± 0.06	0.78 ± 0.29	7.22 ± 2.76	0.18 ± 0.014	4.57 ± 0.018	1.055 ± 0.151	4330 ± 622	MAM
2	Jhura	JFTL2	d4	0.78	0.34 ± 0.03	3.78 ± 0.52	15.35 ± 2.66	1.393 ± 0.009	21.5419 ± 0.0096	3.471 ± 0.164	6207 ± 292	MAM
3	Jhura	JFTL3	d3	1.28	0.42 ± .04	3.890 ± 0.715	17.28 ± 2.494	1.47 ± 0.020	25.533 ± 0.16	3.688 ± 0.176	6924 ± 334	MAM
4	Jhura	JFTL5	d1	2.8	0.36 ± 0.03	4.26 ± 0.8	18.659 ± 3.42	0.398 ± 0.027	22.874 ± 0.147	2.78 ± 0.22	8229 ± 658	MAM
5	Jhura	JFTL6	c4	0.76	0.22 ± 0.02	5.12 ± 0.83	18.423 ± 3.528	1.028 ± 0.061	31.5 ± 0.27	3.63 ± 0.236	8670 ± 568	CAM
6	Jhura	JFTL7	c3	1.43	0.26 ± 0.02	3.62 ± 1.02	14.32 ± 1.61	0.74 ± 0.008	24.779 ± 1.18	2.706 ± 0.188	9159 ± 772	MAM
7	Jhura	JFTL8	c2	0.96	0.44 ± 0.04	3.44 ± 0.68	16.78 ± 4.67	1.12 ± 0.17	31. ± 187 ± 0.884	3.21 ± 0.298	9716 ± 942	CAM

CAM-Central Age Model; MAM-Minimum Age Model; ED-equivalent dose; U-238, uranium; Th-232, thorium; K, potassium.

**Figure 5:**
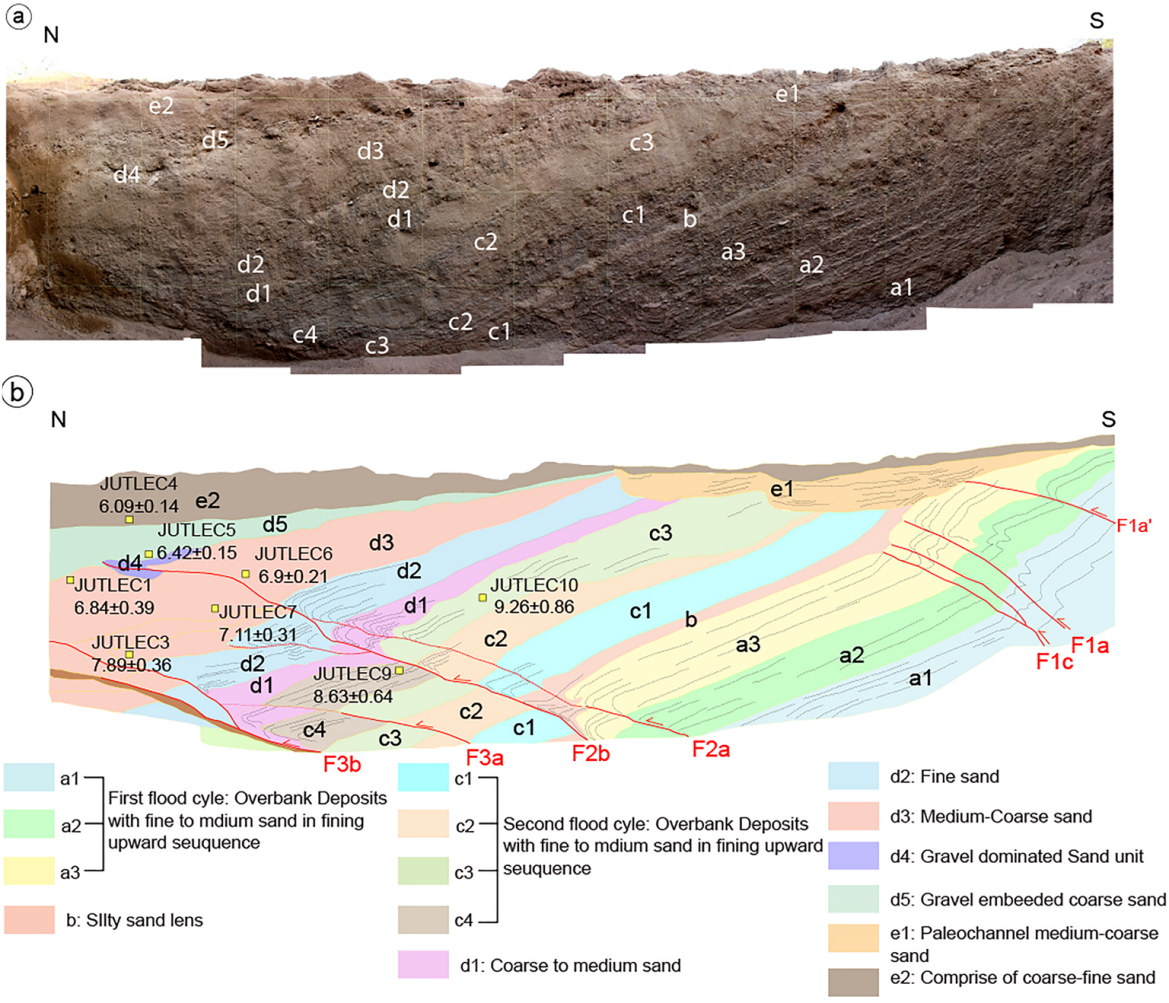
(**a**) Photo-mosaic of the east wall of the Trench-II, grided with 1 × 1 m; (**b**) Trench log of the east wall with fault strands. Location of OSL dated samples are marked by yellow boxes with ages.

*Refer to Fig. 5 and supplementary data Fig. S8 for sample location on the trench wall.

### Kinematic modelling and magnitude computation

To achieve a broader view of the geologic structures and their related deformation, we built a shallow geologic cross-section composing the information acquired in the dug trenches.

We restored the composite section through a trishear kinematic model considering the observable offset and deformation of the stratigraphic units and the associated folding in the hanging wall (Fault Fold code^67,68^). Additionally, the trishear kinematic inversion output also allowed the investigation of the velocity of fault propagation through the overlying stratigraphy since the fold geometry depends on the propagation on the slip ration (P/S value).

To minimize computing time, we performed our search in successive steps: firstly, making evaluations over large regions of values and then focusing over smaller ranges, testing small increments in parameter values such as ramp angle, fault tip position and propagation to slip ratio (P/S). We tuned the trishear parameters manually for the faults characterized by a small amount of displacement and folding. In contrast, for the not fully exposed faults we employed an inverse grid search of the trishear parameters, especially those not directly constrained by the trench exposure. The fold limb has been unfolded by keeping the amount of P/S computed during the previous steps, and we moved the fault ramp angle and tip position to restore the visible horizons to a nearly horizontal geometry. For a complete description of the tested models, the grid search runs, and the ranges of the tested parameters refer to the Supplementary Material (Figs. S4–S7 and Tables S1 and 2).

We finally used the age constrained events, the fault trace length and the dip angle obtained from kinematic reconstruction, the observed and computed slip, the calculated rupture area and a literature seismogenic thickness of 30 km, for computing the expected magnitude through well-known empirical relationships^69,70^.

## Results

### Main morphotectonic features

In the piedmont zone of the JA, three levels of Alluvial Fan Surfaces (AFS 1–3) were identified (Fig. 2). AFS-1 is located on the hanging wall side of the JF, while AFS-2 and AFS-3, along with multiple river terraces, were observed on the footwall side. Only remnants of AFS-2 crop-out at higher level compared to AFS-3 near the Kaila River valley outlet (Fig. 2c). Differentiating AFS-2 from AFS-3 was challenging due to vegetation and small channels, requiring reliance on quantitative 3DA analysis. Additionally, four levels of fluvial fill and cut terraces were observed along the Kaila River in the eastern part of the JA (Figs. 2a, c).

The outcropping JA extends approximately 16 km in the WNW-ESE direction (Fig. 2b). Conversely, the active fault scarp observed only for about 10 km, most likely because of poor preservation and human modifications such as construction of ponds on the upthrown side of a road (Fig. 3b). Notably, the scarp is better preserved where it displaces one of the mapped AFS (Figs. 2b, 3a-b).

The vertical offset (VS) across the ~ 10 km fault scarp north of the JA varies from 4–15 m across different alluvial fan surfaces, peaking near the central part at about 5 km along the profile (Figs. 3c and S2). Additionally, a minimal offset of 0.5–1 m in AFS-3 suggests the presence of a fault splay in the footwall of the main fault in VS profiles FS-2 and FS-4, although this was obscured by agricultural activities (Figs. 3d and S2). The longitudinal swath profile of JA shows a maximum relief of around 300 m above m.s.l., with the SE portion being steeper (Fig. 3c). The comparsion of VS and elevation profiles reveals about 1 km south-eastward shift in the maximum values of the scarp height relative to the topographic peak. The highest topographic points align with the minimum scarp height, followed south-eastward by increasing VS. Finally, both distributions exhibit a right skewness, more pronounced in the VS values (Figs. 3c and S2).

### Trench stratigraphy and structural features

#### Trench-I

A 6-m-long, 2.5-m-deep, and 3-m-wide trench (location: 23°25′40.86" N, 69°36′57.14" E; WGS84; Figs. 3b, 4 and S1b) was excavated at the base of the fault scarp, just ahead of a pond that exposes overturned Tertiary beds.

The trench stratigraphy (Fig. 4) consists of five units of near vertical Tertiary beds (T1–T5) capped by Quaternary deposits (a1’). The T1 unit consists of micaceous sandstone and is separated from unit T2 by a south-dipping reverse fault (57°) flattening upward. Unit T2 is formed by marly clay comprising a heavily fractured siliciclastic layer. The near-vertically dipping T3 unit comprises siltstone crushed due to intense tectonic deformation. The T4 unit comprises ferruginous mudstone, and T5, in the extreme north of the trench, consists of deformed sandstones (Fig. 4). The overlying Quaternary deposits are formed by the older alluvium that belongs to AFS1, dominated by angular pebbles attesting the short-distance sediment transport from a nearby source (Fig. 4).

#### Trench-II

A 12 m long, 2–3 m deep, and 3.5 m wide trench (location: 23°25′41.26"N, 69°36′57.63"E; WGS84; Figs. 3b and S1c). The walls of the excavated trench (Fig. 5) exposed gently dipping layers of unconsolidated sand and gravel, displaced by several south-dipping thrust splays. The exposed succession in the east wall of Trench-II has been divided into five units (labelled from *a* to *e*; Fig. 5b). The sediments composing these units are overbank alluvial deposits emplaced during successive flood cycles, and typically exhibits a fining-upward sequence. In particular, units *a1* to *c* represent an upward fining sequence ascribable to individual flood cycles. The coarser sediments at the bottom consist of matrix-supported fine gravel, gradually grade upward to coarse sand. Unit *c4* is partly eroded on top of the hanging wall of fault F2, indicating an erosive phase postdating *c4* and predating the following depositional cycle. The overlying units *d* and *e* are made of medium sand to silt deposited in an overbank environment. The sequence shows a diverging geometry and host a fault-propagated-fold near the fault tip along with the dragging of units resulting in local recumbent folding (Fig. 5). The fault strands displacing these units show a progressively lower dip moving northward.

The strands F1b and F1c (in the southern portion of the trench) displaced units *a1-a3* and are sealed by unit *b*, whereas the fault strands F2a and F2b deformed units *a1-a3* (the displaced *a1* is not exposed in the trench), *b*, *c1-c4* and *d1-d5*. However, F2b accommodates much more displacement with multiple rupture events as the offset from *a3* to *c3* registers displacement of ~ 60–70 cm compared to ~ 10–20 cm of offset in *d1* to *d4* units. The F2a records a little slip ranging between ~ 5–15 cm. In the southern bottom portion of the trench, the strand F3a marks deformation displacing *c1-c4*, *d1-d3* and *d5* by ~ 60 cm. Deformation along the strand F3b shows a distinct thin layer of mixed material indicative of a shear zone comprised of overturned units dragged during slip along the fault (Fig. 5).

Similarly, the west wall was classified following the same unit code as the east wall (Fig. S8a-b). However, the units with *a* and *d* codes were subdivided into more sub-units, i.e., *a1’-a3* and *d1-d5*, and few units in the west wall are thicker that the correlated counterparts on the east wall (Figs. 5b and S8a-b). As far as the structure is concerned, the west wall has more tilting and warping in the layers than the east wall, and the fault strands are evident, with at least the latest event displacing all the beds by the same amount causing the propagation of the fault at the surface.

#### Trench-III

**Figure 6:**
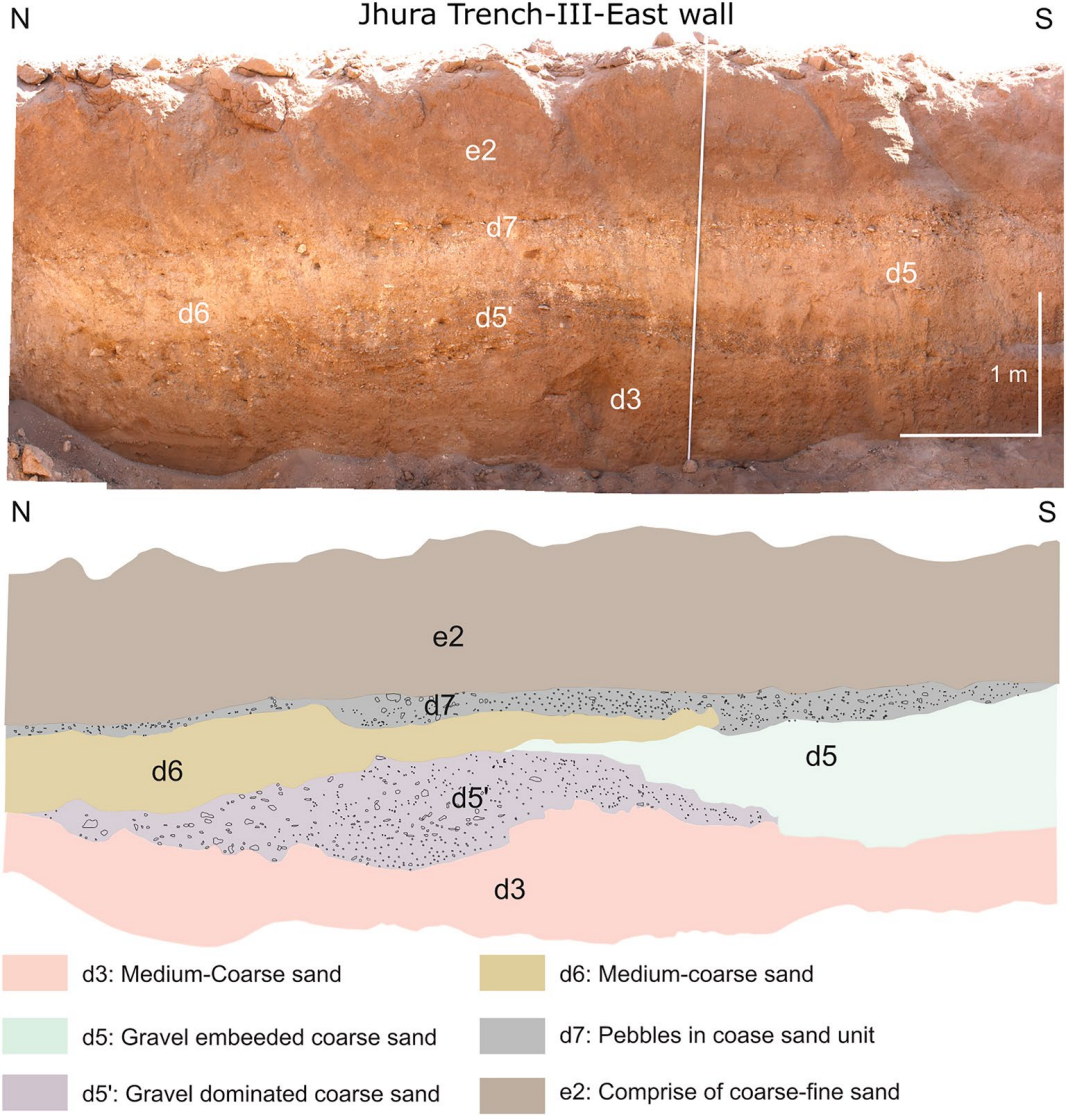
(**a**) Photomosaic of the east wall of Trench-III, displaying negligible deformation; (**b**) trench Log.

The further excavation of Trench-II was obstructed by an underground water pipeline. Therefore, looking for the deformation on fault F3b, Trench-III was excavated next to T-II after leaving a gap 2 m (Figs. 3b and S1d). The excavated trench was 7 m long, 2–3 m deep, and 3 m wide (location: 23°25′41.47"N, 69°36′57.73"E; WGS84; Fig. 3b), and it revealed a thick sequence of overbank deposits, stratigraphically placed on top of unit *d3* (Fig. 6). From the base, a thick sandy layer with medium-coarse gravel intercalations, coded as *d5’*, is partly overlain by the unit *d5* and by another event of overbank deposition (unit *d6*). These units are capped by unit *d7*, a medium to coarse gravel embedded in unconsolidated sand, reflecting a high energy transport. On the top, the continuation of the *e2* layer was laterally followed from Trench-II toward this one (Fig. 6).

The depth of the trench was maintained to 2–3 m to preserve the safety conditions of the working people under unstable trench walls.

#### Trench-IV

This trench was located ~ 70–80 m west of Trench-II (23°25′43.51"N, 69°36′54.72"E WGS84; Fig. 3b), collapsed shortly after the excavation. Initial investigation of the partially cleaned east wall revealed a south-dipping reverse fault, with another fault strand near the base (Figs. S1e and S9). To view it properly, further excavation was planned but led to wall collapse.

**Figure 7:**
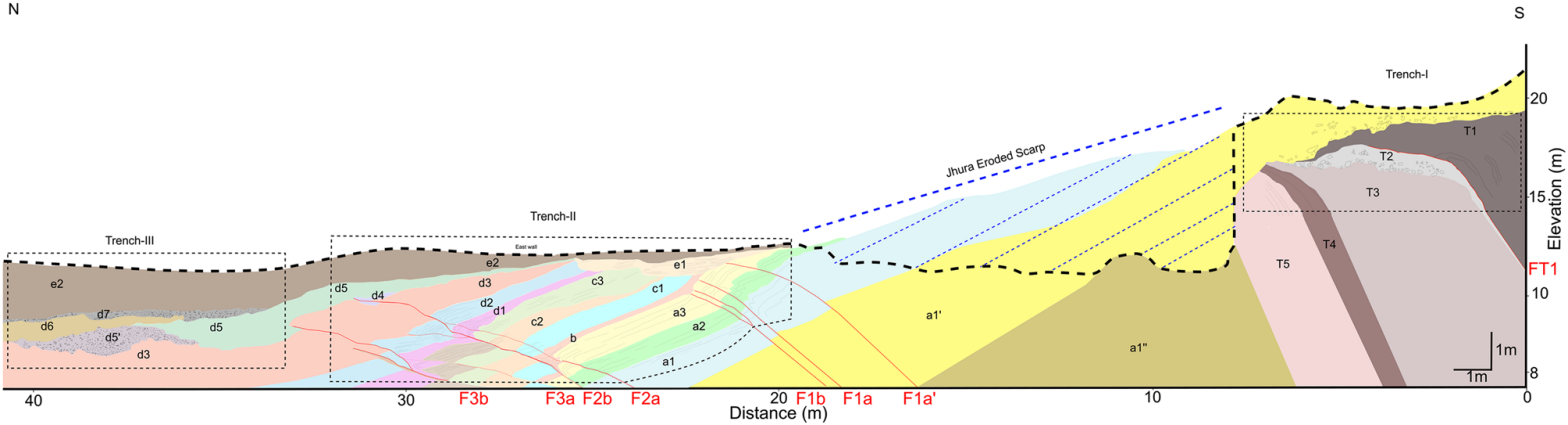
A composite cross-section along the excavated Trenches I, II and III (projected): the trace of the section is indicated in Fig. 3.

An overall view of the fault zone was obtained through a composite section built along the dug trenches (Fig. 7). Later we used this composite trench log to restore the deformation history of the fault across the entire deformation zone.

### Trench retro-deformation

**Figure 8:**
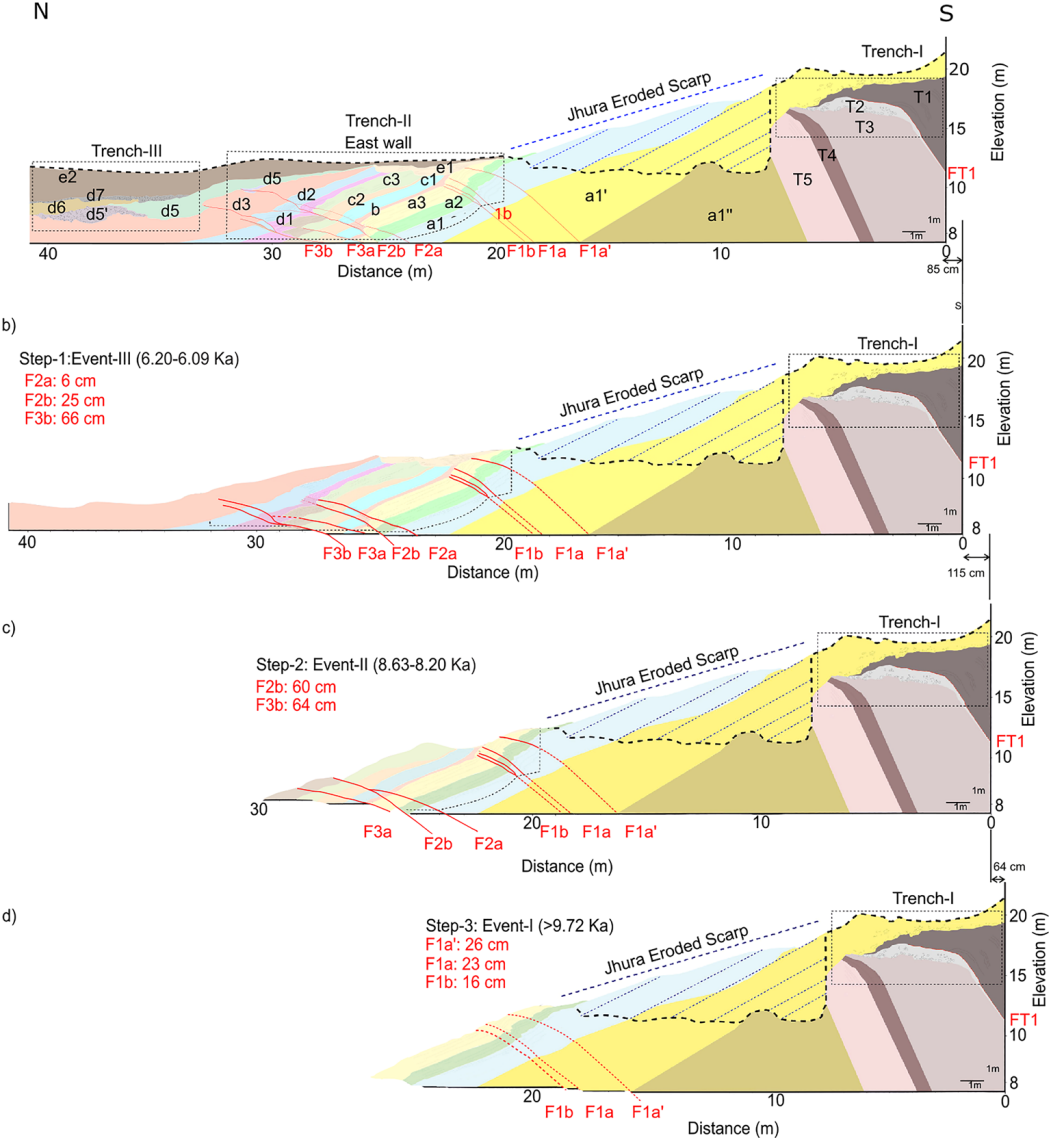
Sedimentary evolution model of the trench composite log. Simple jigsaw progressive retro-deformation of the composite section through the latest three surface-rupturing events: (**a**) Present-day section; (**b**) restoration of Event III: 85 cm of horizontal shortening are distributed among three different fault strands (F2a, 2b and F3b); (**c**) restoration of Event II: 115 cm of horizontal shortening are taken up by the F2b and F3b fault strands; (**d**) restoration of Event I: 64 cm of horizontal shortening is estimated for this event along F1a’, F1a and F1b fault strands. Figure generated with FaultFold v.7.2.0 (http://www.geo.cornell.edu/geology/faculty/RWA/programs/faultfoldforward.html).

**Figure 9:**
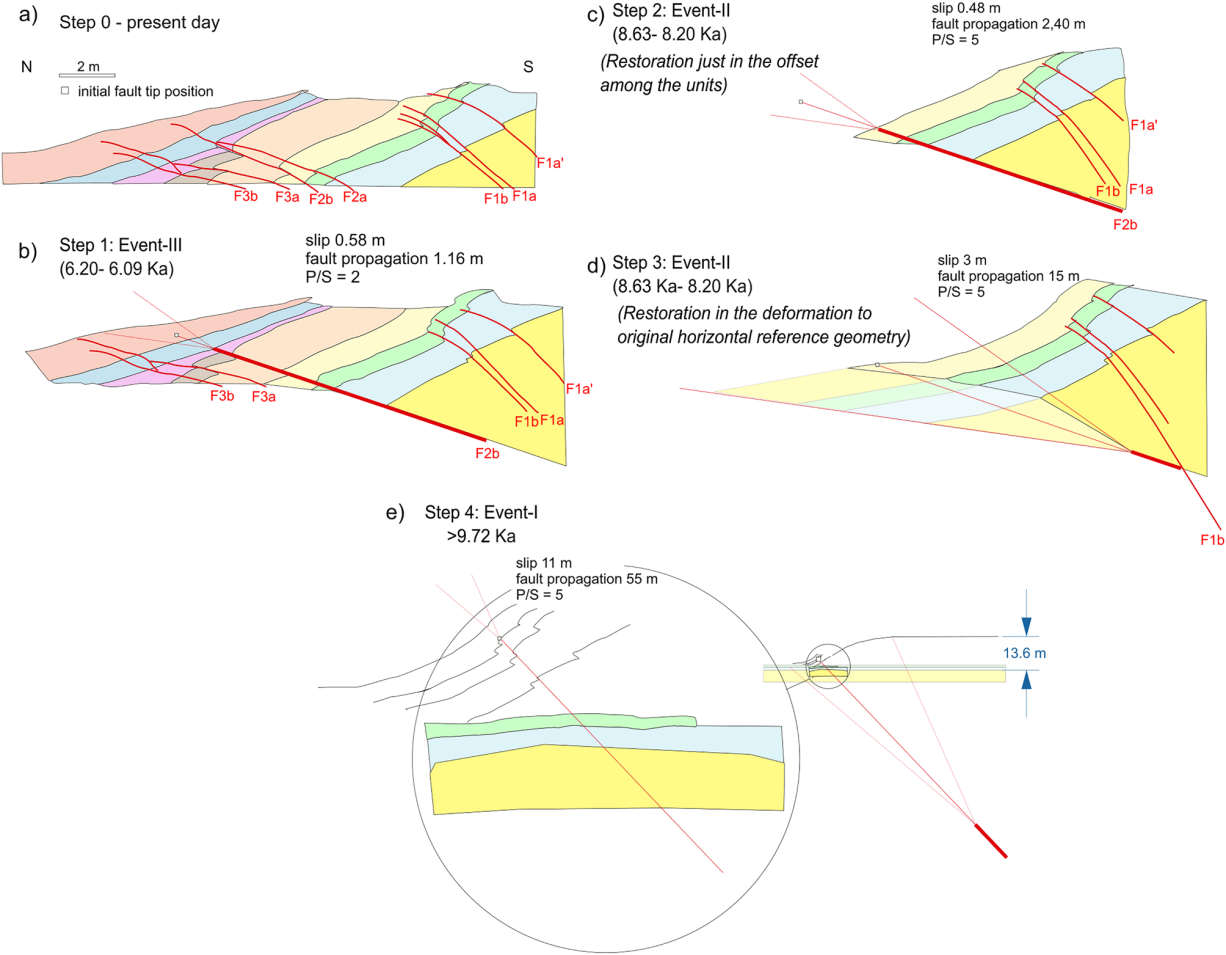
Progressive restoration of the fault-propagation fold across the composite trench, adopting a trishear kinematic modelling: (**a**) to (**d**) are the restoration of the latest events, here modelled as single fault ruptures; (**e**) restoration of the fault-propagation fold accounting for the cumulated deformation up to ca. 9.72 Ka BP and building the fault scarp at this location. Figure generated with MOVE suite software provided by Midland Valley (https://www.petex.com/pe-engineering/move-suite/).

The initial step of the trishear retro-deformation started from the present geometry, reconstructed in the composite trench log (Fig. 7). We first simply applied a retro-deformation of the offsets identified in the trenches, obtaining 0.85 m of horizontal shortening for Event III, and 1.15 m and 0.64 m for Event II and I, respectively (Fig. 8). For the Event I and unit *d3* (Step 0, Fig. 9a), the retro-deformation performed through an iterative change approach in the P/S value resulted in an optimal P/S = 2 and obtained a fault propagation of 1.16 m with a slip of 58 cm (Step 1; Fig. 9b). Considering Event II, after testing more than 100,000 models, we first restored the offset measured in the different units, obtaining 48 cm of slip (Step 2; Fig. 9c), then unfolded Unit *a3* to restore the F2 fault-propagation folding during Event II (Step 3; Fig. 9d). The outcoming parameters are a P/S = 5, and a total slip during this last phase of fault growth of ca. 3.5 m. These parameters imply a fault propagation of ca. 17.4 m, reaching a depth of ca. 7 m below the top of the trench where F2 intersects F1 (Fig. 9d). Finally, the fold front limb, that is representative of the cumulated, long-term activity of the JF, has been unfolded through the trishear restoration of Fault F1, resulting in a total slip of 11 m and propagation of the fault tip of 55 m, reaching a depth of ca. 65 m (Step 4; Fig. 9e).

This last retro-deformation includes Event I, and probably other older events with no dated units in the trenches that can help in constraining the age of the events.

### Age constraints of paleo-earthquakes

We dated the logged units from fifteen out of twenty-three collected samples. The dated samples provide critical information on the sequence of paleo-events from this area. The OSL ages derived from both the east and the west walls of the Trench-II, combined with the stratigraphic interpretation and retro-deformation, indicates at least three significant Holocene paleo-earthquakes along this segment of the KMF (Figs. 5, S10–S13 and Table 1). However, the older event may be a compound of multiple events, as suggested by the retro-deformation analysis (refer Sect. 4 in Supplementary Data).

Based on the cross-cutting relationships, slip and folding observed in the exposed sedimentary units on the east wall of the Trench-II, as well as their OSL ages, we infer that the surface faulting events were caused by a slip on the JF (Figs. 5, 8 and 9). The oldest Event I, along with potentially earlier events that caused deformation and folding and displacement along the F1a-c strands (Fig. 5b). The Event I folded and displaced units *a1-a3*, followed by a phase of erosion and deposition of unit *b*. The penultimate Event II occurred after the deposition of units *c3-c4* and before the deposition of unit *d1*. Considering ages only from the east-wall, it is suggested that Event II occurred between 8.63 ka and 7.11 ka BP (Figs. 5 and 8c). Further, the deposition continued from unit *d1* to unit *d5*. Unit *d1* deposited after the erosion of Unit *c4* in the hanging wall of fault F2b. The latest Event III (Most Recent Event—MRE) took place after the deposition of unit *d5* and before the deposition of unit *e2* (Figs. 5 and 8b). MRE was registered by F2b with a minor slip along F2a, as well as along F3a strands. But most likely, F3a and F3b moved displacing the younger sucession (Unit d5) during Event III (Fig. 7).

Based on the OSL ages from the east wall we suggest that Event III (MRE) occurred between 6.42 ka and 6.09 ka BP (Fig. 5a,b). Conversely, the stratigraphy of the west-wall highlights the occurrence of just two events because the deformation was taken up mostly by folding, rather than faulting (Fig. S8). Due to erosion of the upper portion of unit *c2* followed by capping (deposition) of unit *e1*, there is no reliable constraints on the amount of offset for the younger units. Further, the folding associated with faulting prevented us identifying possible successive events on F1W. Therefore, the F2aW and F2bW strands were considered for bracketing the age of the events. Considering the ages from the west wall, we infer that the penultimate Event II occurred between 8.67 ka and 8.23 ka, and the MRE in between 6.21 ka and 4.33 ka (Fig. S8). The MRE led to the reactivation of F1w and activation of new fault strands, which in our log are named F2aW and F3W. The deformation on the east-wall of Trench-II, suggests that the number of events might be more than three. Furthermore, the OSL ages from the west-wall were also considered to better constrain the events. With the similar identification of layers based on lithologies, the Event-II was bracketed between 8.63 ka and 8.23 ka and subsequently the Event III (MRE) between 6.20 ka and 6.09 ka (Figs. 5b and S8).

### Expected fault magnitude

The fault parameters derived from field mapping (anticline and fault scarp length), and the data retrieved from the paleoseismological trenches about the displacements related to the paleo Events II and III are listed in Table 2. This data was used for calculating the expected moment magnitude of future earthquakes generated by the JF. For these calculations we applied empirical relationships from Wells and Coppersmith (1994, WC-94) and Leonard (2014, L-14), considering the mapped lengths of the anticline and fault scarp as possible extents of surface faulting events. We excluded Event I from the calculation because of the limitation with the ages and possible cumulative deformation as evidenced in the retro-deformation.

**Table 2:** Estimations of magnitude derived from varying input data for the most recent surface-rupturing earthquakes, Event-II and Event III, observed in the Jhura Trench-II (WC94: Wells and Coppersmith, 1994; Leonard, 2014).

Fault	Measured parameters	Regression relation	Input value	Moment magnitude	Reference
Event II and III on F1 and F2, respectively	Jhura Anticline length (Mapped)	Surface rupture length	16 km	6.47 ± 0.29	WC-94
	Fault scarp length	Surface rupture length	10 km	6.22 ± 0.27	WC-94
	Jhura Anticline length (Mapped)	Surface Rupture Length	16 km	6.31	L-14
	Fault scarp length	Fault Rupture Length	10 km	6.11	L-14
	Dislocation in trench of Event III (MRE)	Maximum displacement	0.58 m	6.42 ± 0.13	WC-94
	Dislocation in trench of Event II	Maximum displacement	0.48 m	6.38 ± 0.14	WC-94
	Dislocation in trench of Event III (MRE)	Displacement	0.58 m	6.00 ± 0.16	L-14
	Dislocation in trench of Event II	Displacement	0.48 m	5.84 ± 0.16	L-14
	Dislocation in trench of Event II	Maximum displacement	3.48 m	6.76 ± 0.18–7.56 ± 0.16	WC-94–L-14

Considering the length of the anticline with WC-94 and L-14 methods, the estimated magnitude ranges between 6.3 and 6.5. For the fault scarp’s length, magnitudes are slightly lower, from 6.1 to 6.2. If we consider dislocations from Events II and III as average displacements, magnitudes range from 5.8 to 6.4. These calculated magnitudes, based on fault length and event offsets, are comparable within uncertainties, indicating the fault's consistent behavior in the long- and short-term. Conversely, using the ca. 3.5 m slip derived from unfolding of the deformed units associated to Event II, would yield a magnitude range between 6.8 and 7.6 (Table 2). This higher estimate seems unrealistic, because according to L-14 empirical relationship a fault over 50 km long would be required to generate an earthquake with magnitude between Mw6.8 and 7.6, contradicting the 16 km length of the mapped anticline.

## Discussion

### Short-term and long-term effects of surface rupturing

We found evidence of at least three surface-rupturing earthquakes along the JF, a branching out fault of the KMF system that borders the southern portion of the Great Rann of Kachchh. In each trench excavation, the one excavated across the fault scarp to the other two dug further ahead in the foreland, it was possible to observe the foreland propagation of the deformation, characterized by a reduction in the dip-angle of the fault splays (Figs. 4, 5, 6 and 7). Moreover, the stratigraphic logs and OSL ages suggest that each earthquake caused a deformational shift towards north in the Banni Plain, resulting the activation of new fault splays and occasionally the reactivation of older fault strands. The total measured slip is approximately 11 m, leading to a cumulative uplift of 13.6 m, which closely matches the observed maximum fault scarp height of 13.98 m (Fig. 3d; Profile FS-3).

Topographic and paleoseismic investigations on the JF revealed the evidence of tectonic activity during Holocene. The similarity between the height of the fault scarp and the relief wavelengths of the anticline suggests that both long-term topography and short-term deformation are driven by the same processes, indicating that the topography closely reflects cumulative deformation on fault (Fig. 3c). Despite observing a right skewness in the VS distribution—potentially due to factors like fault complexity, lithological differences, fault slip kinematics, slip distribution, or erosion—the alignment of the VS distribution with the anticline's topography highlights a substantial continuity between the short-term and the long-term fault behaviour^71,72^.

### Updating seismic catalogue

Previous studies in the Kachchh basin have identified multiple paleo-earthquakes. Rajendran and Rajendran (2001)^9^ based on paleo-liquefaction features linked an event between 875–1035 CE near the 1819 source to historical accounts of an 11th-century earthquake that destroyed Brahminabad north of the ABF scarp. Paleoseismological studies on the secondary ruptures and liquefaction around 2001 Bhuj epicenter suggested two earlier events around 4000 and 9000 years ago^33^. Trench studies on the SWF identified three prehistoric earthquakes: one before 6972–6666 BP, another between 4753–4149 BP, and the most recent between 2109–1913 BP ^30^..

Kothyari et al. (2021)^34^ discovered four Late-Middle Holocene events from trenching on the KMF’s western segment, occurring between 2000–2900 years BP, 3500–3800 years BP, 4000–4600 years BP, and the most recent between 890–1800 years BP, possibly associated to the 11th-century event. At the archaeological site of Dholavira, close to Khadir Island, a settlement had existed for about 1500 years (3450–4950 years BP), and an earthquake damaged it sometime between 4150–4450 years BP (Fig. 1 for location)^73^. Thus, this event can be correlated with an event between 4753–4149 BP identified along the SWF, as well as with the one identified in the liquefaction study from the 2001 epicentral area^30,33^ (Fig. 1).

Our study adds three new surface rupturing events: Event I before 9.72 Ka BP; Event II between 8.63–8.20 ka BP; and Event III (MRE) between 6.20–6.09 ka BP, expanding the paleo-event catalogue. Estimated magnitudes from offsets in trenches range between Mw 6.0–6.4 for Event III, and Mw 5.8–6.4 for Event II. However, the 3.5 m slip obtained from unfolding the Event II implies a 50 km long rupture, which is not observed from the morphometric expression of the fault scarp bordering the JA. Hence, the modelling result poses an open question regarding the possibility that the deformation observed in the trenches may have resulted from a cumulative slip of more than one earthquake, or that the earthquake ruptured multiple segments of the KMF. On the contrary, Event III correlates well with the mapped fault scarp length and the observed offset, most likely confined to JF.

### Recurrence interval in intraplate regions

Many thorough paleoseismic studies have been conducted in different continental interiors, including the Rhine graben in Germany, the Bohemian Massif in Czech Republic, Central Asia, Australia and the New Madrid in the USA, have identified the evidence of paleo-earthquakes having recurrence intervals (RI) from a thousand to over ten thousand years^74–86^. Based on the evidence of paleo-earthquake identified in our trenches as well as the previous paleoseismic studies (Table 3), we suggests that the Kachchh is one of the most seismically active intraplate regions in the world having RI of about 2000 years^9,33–35^. This is also consistent with the short-term convergence rate of ~ 3 mm/year reported from the continuous GPS measurement from the Kachchh and its adjoining areas^49^.

**Table 3:** List of paleoearthquakes occurred in the Kachchh region and identified by means of paleoseismological and archeoseismological studies. ABF: Allah Bund fault; KMF: Kachchh mainland Fault; SWF: South Wagad Fault.

Fault	Age	Evidence	Reference
ABF (?)	Eleventh century	Historical sources on the destruction of the village of Brahminabad	R&R-2001^9^
ABF	1075–915 BP	Paleo-liquefaction features	R&R-2001^9^
KMF	1800–890 BP	Paleoseismological trenching	K&2021^34^
SWF	2109–1913 BP	Paleoseismological trenching	M&2017^30^
KMF	2900–2000 BP	Paleoseismological trenching	K&2021^34^
KMF	3800–3000 BP	Paleoseismological trenching	K&2021^34^
2001 Bhuj earthquake source (?)	4000 BP	Paleoseismology on 2001 secondary ruptures and liquefaction features	R&2008^33^
?	4450–4150 BP	Archeoseismological evidence of the damaging of Dholavira	J&B1994^72^
KMF	4600–4000 BP	Paleoseismological trenching	K&2021^34^
SWF	4753–4149 BP	Paleoseismological trenching	M&2017^30^
SWF	6972–6666 BP	Paleoseismological trenching	M&2017^30^
2001 Bhuj earthquake source (?)	9000 BP	Paleoseismology on 2001 secondary ruptures and liquefaction features	R&2008^33^

Considering the return period and paleoseismological results, it is quite logical to suggest that the JF portion has temporal earthquake clustering. This includes one or more events nucleated before 9.72 ka, as observed from a cumulative fault scarp. Notably, Events II and III occurred within a relatively shorter span of 2000 years, and since then no earthquake has been experienced as observed from the trench investigation. Possibly, this can be explained hypothesizing two scenarios: (1) the stress may have been transferred to the neighbouring segments that generated younger events, or (2) more recent seismic events may have not been identified in our trenches due to removal of sediment succession AFS2 by erosion. If these scenarios are not true, then possibly the stress accumulated along JF since the last event hints towards having a major earthquake in near future.

## Conclusion

This study provides key insights about the seismo-tectonics of the Kachchh region, uncovering significant surface faulting paleo-earthquakes along the Jhura Fault (JF)—a branching out fault of the Kachchh Mainland Fault (KMF), with magnitude ranging from Mw 6.0–7.5, in the vicinity of the 2001 Bhuj earthquake. With a RI of ~ 2000 years the Kachchh region can be placed as one of the most active intraplate regions in the world. The 10–16 km long active fault scarp along Jhura Fault (JF) bordering the Jhura Anticline (JA) and varying offsets observed in the paleoseismic trenches are indicative of coseismic surface ruptures associated with major paleo-earthquakes triggered by stress transfer across the adjacent segments of KMF.

Given the above insights, we emphasize that further comprehensive research on long-term deformation is required to understand the tectonic behaviour of active faults and associated fault segments of KMF.

### Supplementary Information

Supplementary Information.

## Data Availability

The dataset, involving images and mosaics of the trenches along with Optically Stimulated Luminescence (OSL) data and curves, and the results of Inverse grid search from Kinematic restoration, are provided in the supplementary sheet. The remotely sensed datasets (CARTOSAT-I, 2005–2006) can be accessed from the Indian government web portal: https://bhuvan.nrsc.gov.in/home/index.php. Historical and instrumental earthquakes were gathered from USGS: https://earthquake.usgs.gov/earthquakes/search/; ISC: http://www.isc.ac.uk/iscbulletin/search/catalogue/; and from the Institute of Seismological Research (ISR), Gandhinagar catalogues. The focal mechanisms are from the Global CMT Catalogue: https://www.globalcmt.org/. The stress indicators are from the World Stress Map (WSM) project and can be accessed at https://www.world-stress-map.org/. All intermediate data processing and calculations generated and analysed during the current study are available from the corresponding author upon request.
