# Supplementary material for: Holocene surface-rupturing paleo-earthquakes along the Kachchh Mainland Fault: shaping the seismic landscape of Kachchh, Western India
Source: Sci Rep. 2024 May 21;14:11612. doi: 10.1038/s41598-024-62086-z (PMC11109187; doi:10.1038/s41598-024-62086-z)
Supplement: Supplementary file 1 — Supplementary Information. [file 41598_2024_62086_MOESM1_ESM.docx]

***Supplementary Data***

## *for*

**Holocene Surface-Rupturing Paleo-earthquakes along the KMF: Shaping the Seismic Landscape of Kachchh, NW India**

**Javed N. Malik^1*^, Eshaan Srivastava^1,2^, Mahendrasinh Gadhavi^3^, Franz Livio^4^, Nayan Sharma^1^, Shreya Arora^5^, Nicolò Parrino^2^, Pierfrancesco Burrato^6^, Attilio Sulli^2^**

^1^ Active Tectonics and Paleoseismology Laboratory, Department of Earth Sciences, Indian Institute of Technology Kanpur, Kanpur 208016 Uttar Pradesh, India. Ph. +91-512-2597723; +91-9956300101.

Emails: [javed@iitk.ac.in](mailto:javed@iitk.ac.in) *Corresponding author; [eshaan@iitk.ac.in](mailto:eshaan@iitk.ac.in); [nayansh@iitk.ac.in](mailto:nayansh@iitk.ac.in)

^2^ Dipartimento di Scienze della Terra e del Mare, Università degli Studi di Palermo, Palermo, Italy

Email: [eshaan.srivastava@unipa.it](mailto:eshaan.srivastava@unipa.it) ; [nicolo.parrino@unipa.it](mailto:nicolo.parrino@unipa.it); [attilio.sulli@unipa.it](mailto:attilio.sulli@unipa.it)

^3^Civil Engineering Department, L. D. College of Engineering, Ahmedabad, Gujarat, India.

Email: [msgadhavi@ldce.ac.in](mailto:msgadhavi@ldce.ac.in)

^4^Università degli Studi dell’Insubria, Dipartimento di Scienza ed Alta Tecnologia, Via Valleggio, 11, 22100, Como, Italy. Email: [franz.livio@uninsubria.it](mailto:franz.livio@uninsubria.it)

^5^Department of Earth and Climate Sciences, Bates College, Lewiston, ME, USA.

Email: [sarora@bates.edu](mailto:sarora@bates.edu)

^6^Istituto Nazionale di Geofisica e Vulcanologia, Rome, Italy

Email: [pierfrancesco.burrato@ingv.it](mailto:pierfrancesco.burrato@ingv.it)

1. **Paleoseismic trenching:** In total 04 trenches (Trench-I to IV) were excavated across frontal deformational zone of the Jhura Anticline (JA) to confirm the deformation along KMF


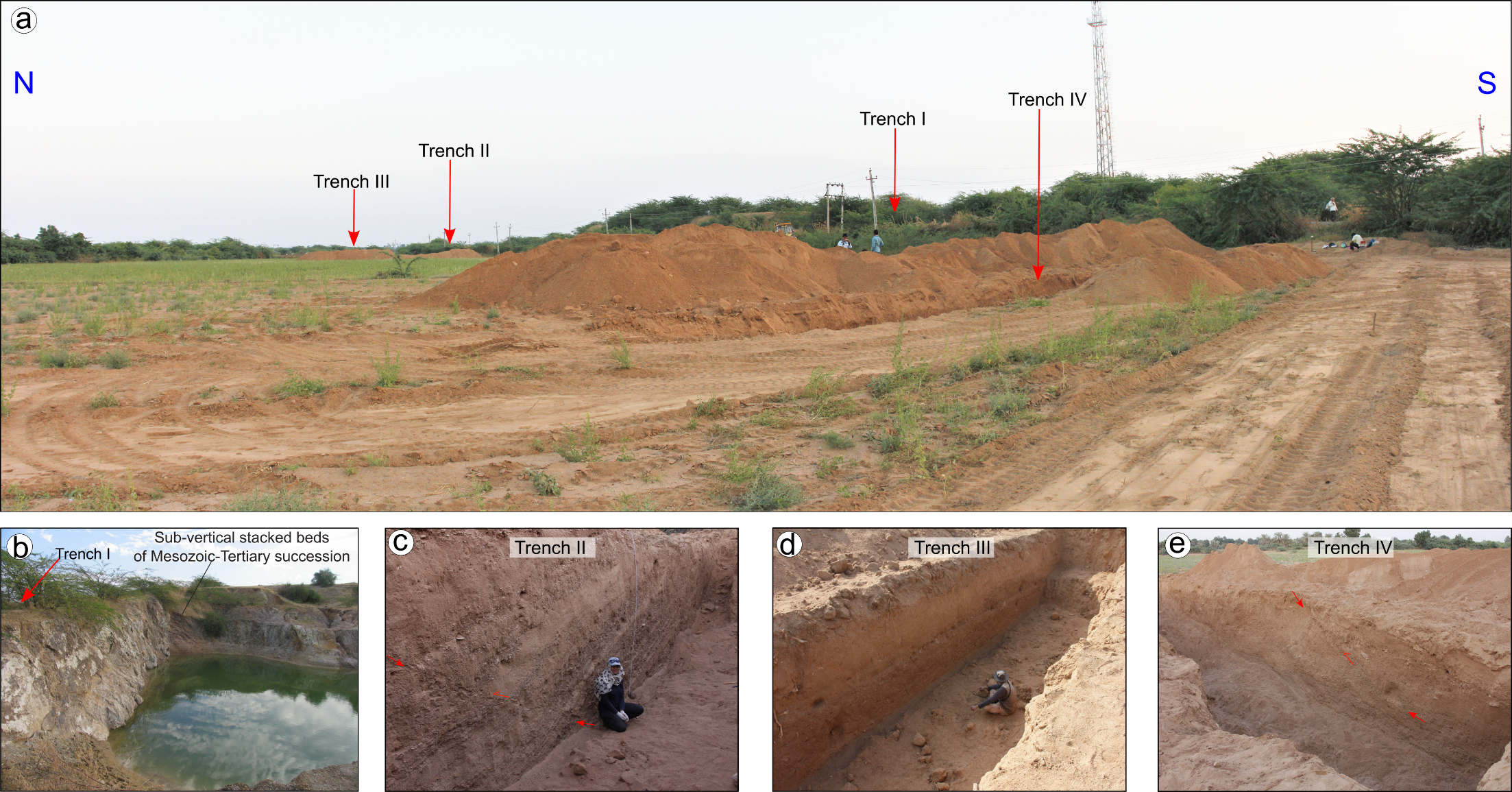


**Figure S1:** (a) A broad view of the dug trenches: (b), Trench-I; (c), Trench-II; (d), Trench-III; (e) Trench-IV, that was excavated parallel to the Trench-II, about 50 m west, and soon collapsed after excavation.

1. **Fault Scarp Profiling:**


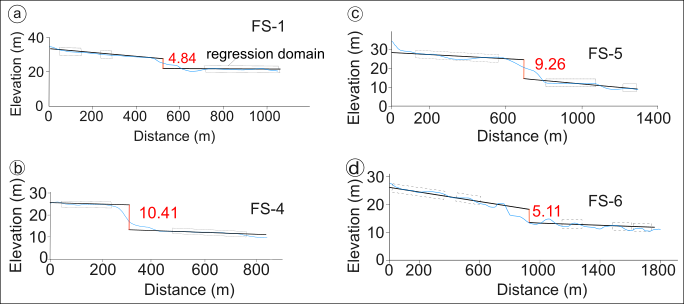


**Figure S2:** Topographic profiles across the Jhura fault scarp, measured on a 3 m resolution CARTOSAT-I DSM as showcased in Figure 3a. figure generated from MATLAB v.R2023a software (<https://www.mathworks.com/products/new_products/release2023a.html>) and Inkscape v. 1.3.0 (<https://inkscape.org/>).

1. **Geophysical Survey:**

The underlying geology surrounding the fault scarp zone of Jhura Dome was investigated using a ground penetrating radar (GPR) scan^1–3^. We observed numerous profiles over 0.5–1m high E–W (strike direction) hitting at terrace T3, which is close to the fault scarp, using a GSSI 200 MHz shielded antenna (<https://www.geophysical.com/antennas>) along with SIR-3000 controller (<https://www.geophysical.com/products/sir-3000>). Due to good lithological contrast, which enables us to pinpoint the precise fault location, several measurements with the following parameters were made: 512 samples/scan, 16 bits/sample, 64 scans/sec, and 100 scans/m ^4^. The near-surface stratigraphy is distorted, and the fault plane exhibits discontinuous georadar reflections. There are south-dipping fault strands because of the reflection (Fig. S3). We processed the GPR profiles in RADAN-7 software (<https://www.geophysical.com/software>) with the following steps in sequence: (a) time zero correction; (b) noise band removal; (c) application of the stacking and infinite impulse response (IIR) filters; (d) adjusting the range gain; and (e) surface normalization. The GPR profile showcased near-surface deformed stratigraphy with discontinuous georadar reflections along the fault strands. We interpret multiple fault strands with high- to low-angle thrust faults, dipping towards the south between 1.0 and 7.0 m (Fig. S3).


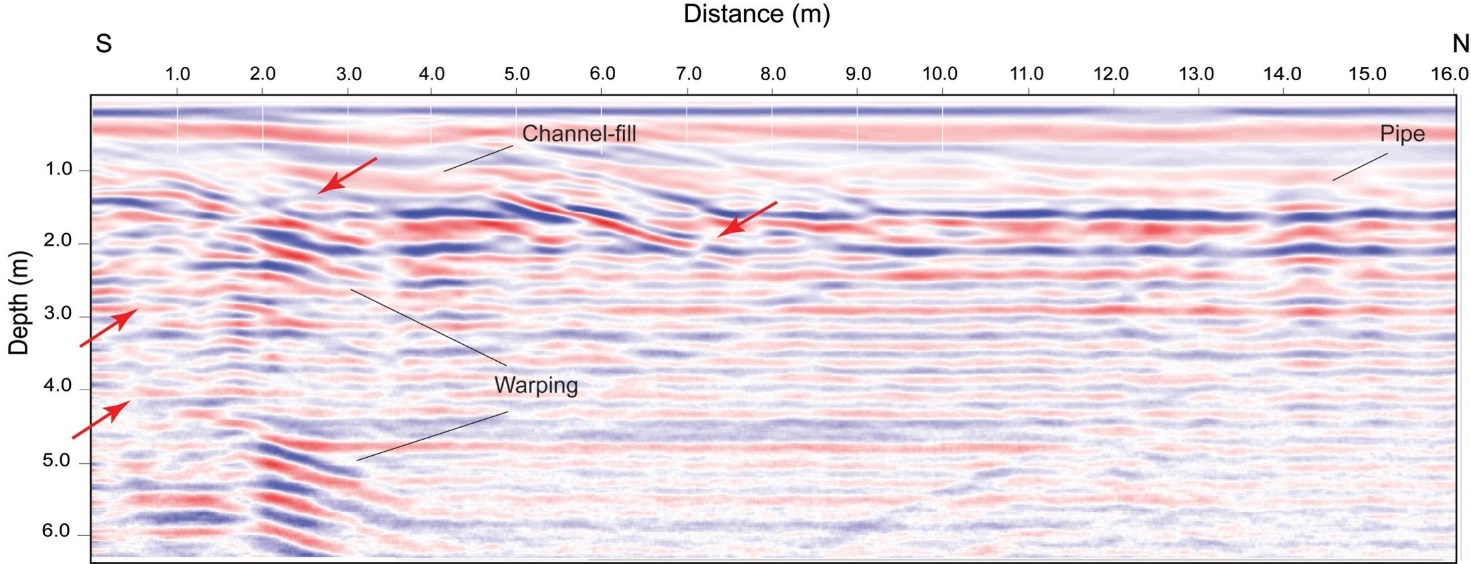


**Figure S3:** Ground Penetrating Radar (GPR:) profile showcasing the deformation in the subsurface of Terrace T3 on the left bank of the Kaila River, just ahead of the identified fault scarp. This resulted in a successful trench at that location, as one can see figure. 5, but confined only up to the first 3 to 3.5 meters due instability of unconsolidated sediment. Figure generated with GSSI 200 MHz (<https://www.geophysical.com/antenna/>) and RADAN-7 software (<https://www.geophysical.com/software>)

**4: Inverse grid search results from Kinematic restoration**

We performed progressive restoration of the F2 fault by means of a grid search inversion approach applied to the trishear kinematic model ^5,6^.

Out of the six possible parameters to be inverted (i.e., ramp angle, trishear angle, propagation on slip ratio P/S, slip, X and Y of the fault tip position), only the trishear angle, P/S and slip have been considered, since the other are well constrained by the present-day trench exposures.

*Restoration of d3 horizon*

Firstly, during a first run of the inversion, we searched over a wide range for parameters to be tested (Table S1). This resulted in ca. 20.000 tested models.

**Table S1:** Grid search parameters for Run 1; minimum, maximum values and the step interval among values are reported, together with the values obtained for the best fit model.

| **RUN 1** | **min** | **max** | **step** | **best** |
| --- | --- | --- | --- | --- |
| *Ramp Dip^0^* | 18 | 22 | 1 | 18 |
| *P/S* | 0 | 6 | 1 | 6 |
| *Trishear Angle^0^* | 10 | 60 | 5 | 25 |
| *Slip (cm)* | 0 | 200 | 5 | 106 |
| *Tip X (cm)* | 2900 | 3000 | 5 | 2910 |
| *Tip Y (cm)* | 160 | 170 | 5 | 160 |

Ramp angle is not that effective in determining the final model performance; nonetheless values close to those ones observed in the exposed trench (i.e., ca. 18^o^) show a best performance (Fig. S6). The tested models showed that a fast propagation of the fault (i.e., P/S > 3) is necessary in order to optimally restore the original horizon geometry (Fig. S7).

**Figure S4:** Run1 - frequency distribution of the Chi squared score obtained for each of the tested values for ramp angle; each boxplot shows the average (line), 2 sigma (boxes) and 3 sigma intervals.

**Figure S5:** Run 1 - frequency distribution of the Chi squared score obtained for each of the tested values for P/S; each boxplot shows the average (line), 2 sigma (boxes) and 3 sigma intervals.

Considering that both ramp angle and P/S show an optimum chi squared score at the edge of the tested range, we performed another run, enlarging the search window for the tested parameters and centering the search window at the best model obtained during Run 1 and reducing the step interval in order to obtain a fine tuning of the inverted parameters (Table S2). This resulted in more than 70.000 tested models.

**Table S2:** Grid search parameters for Run 2; minimum, maximum values and the step interval among values are reported, together with the values obtained for the best fit model.

| **RUN 2** | **min** | **max** | **step** | **best** |
| --- | --- | --- | --- | --- |
| *Ramp Dip^0^* | 16 | 20 | 1 | 18 |
| *P/S* | 0 | 10 | 1 | 5 |
| *Trishear Angle^0^* | 20 | 40 | 1 | 25 |
| *Slip (cm)* | 0 | 200 | 5 | 106 |
| *Tip X (cm)* | 2900 | 3000 | 5 | 2900 |
| *Tip Y (cm)* | 160 | 170 | 5 | 160 |

The second run of the grid search inversion confirmed the overall distributions of errors observed during Run 1. Ramp angle is consistently showing low value of errors between 16^o^ and 18^o^ with a progressive increase of errors at increasing angles (Fig. S8). Additionally, we found a clear minimum value of chi-squared score between five and eight (Fig. S9), clearly pointing that a fast fault propagation is necessary to optimally restore the original horizon geometry.

**Figure S6**: Run2 - frequency distribution of the Chi squared score obtained for each of the tested values for ramp angle; each boxplot shows the average (line), 2 sigma (boxes) and 3 sigma intervals.

**Figure S7:** Run 2 - frequency distribution of the Chi squared score obtained for each of the tested values for P/S; each boxplot shows the average (line), 2 sigma (boxes) and 3 sigma intervals.


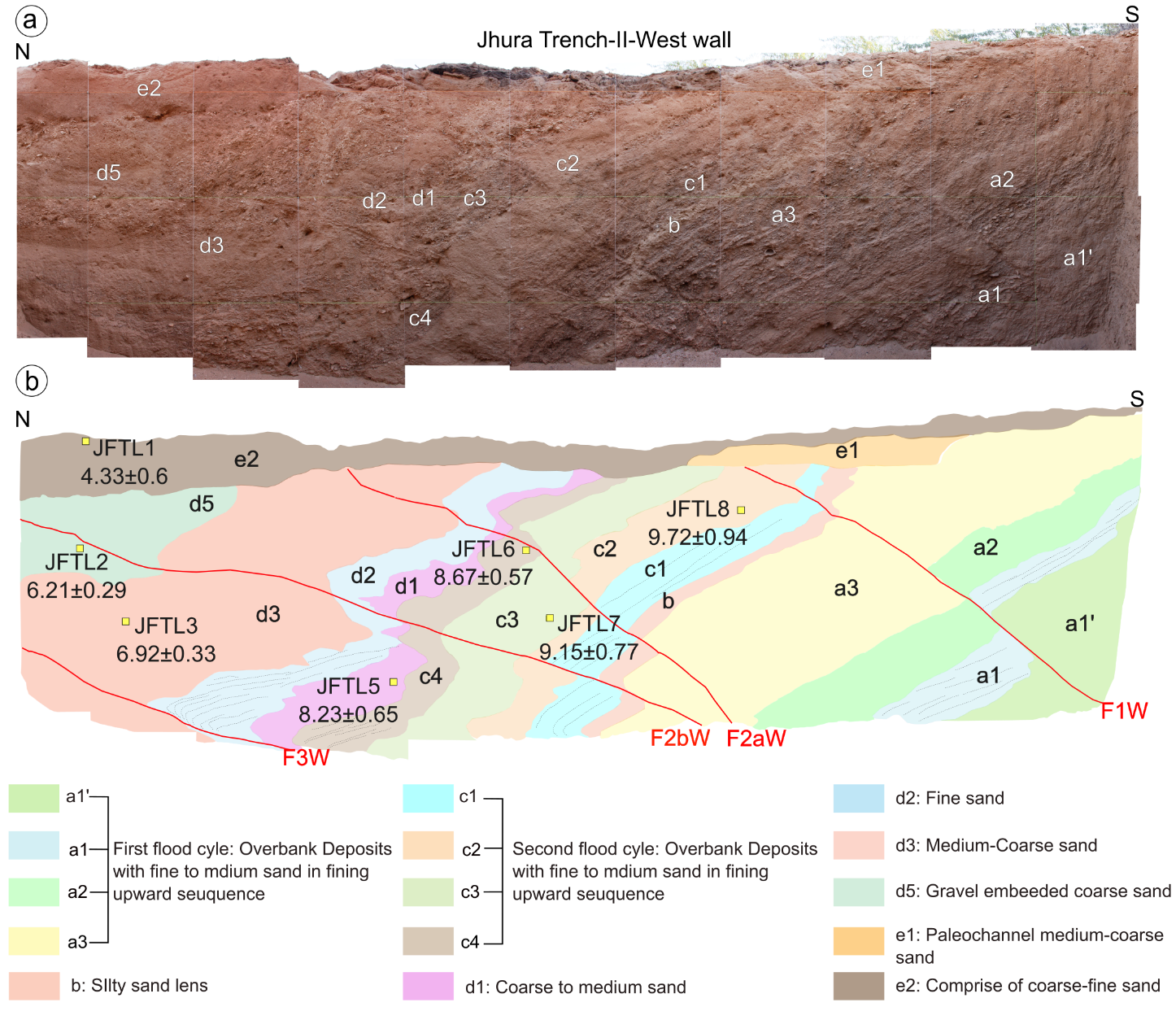


**Figure S8:** (a) The Trench-II west wall mosaic, also grided with 1x1 m, reports more inclination in the beds, which are correlated with the opposite wall, dissected by four fault strands dipping south; (b) litho-log with the main lithological units, identified faults and ages of samples from OSL.

**
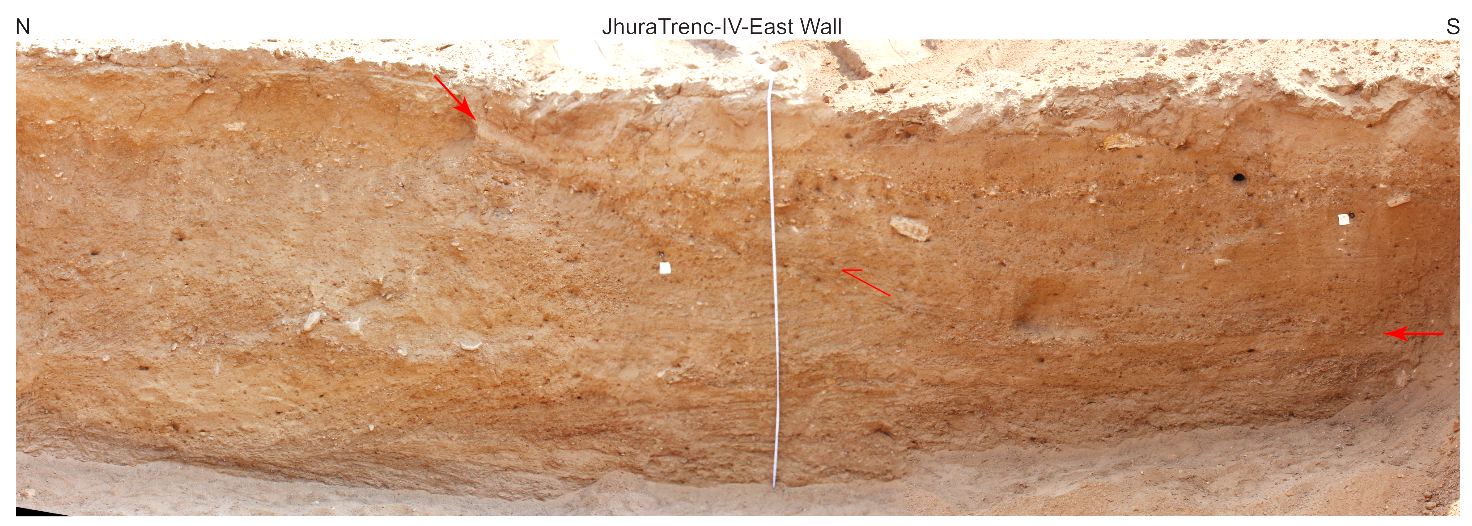
**

**Figure S9:** The collapsed Trench-IV excavated in parallel with Trench-II to look for the lateral presence of deformation near the piedmont zone of Jhura anticline. It shows a listric thrust fault dipping south.

**5: OSL data and curves**


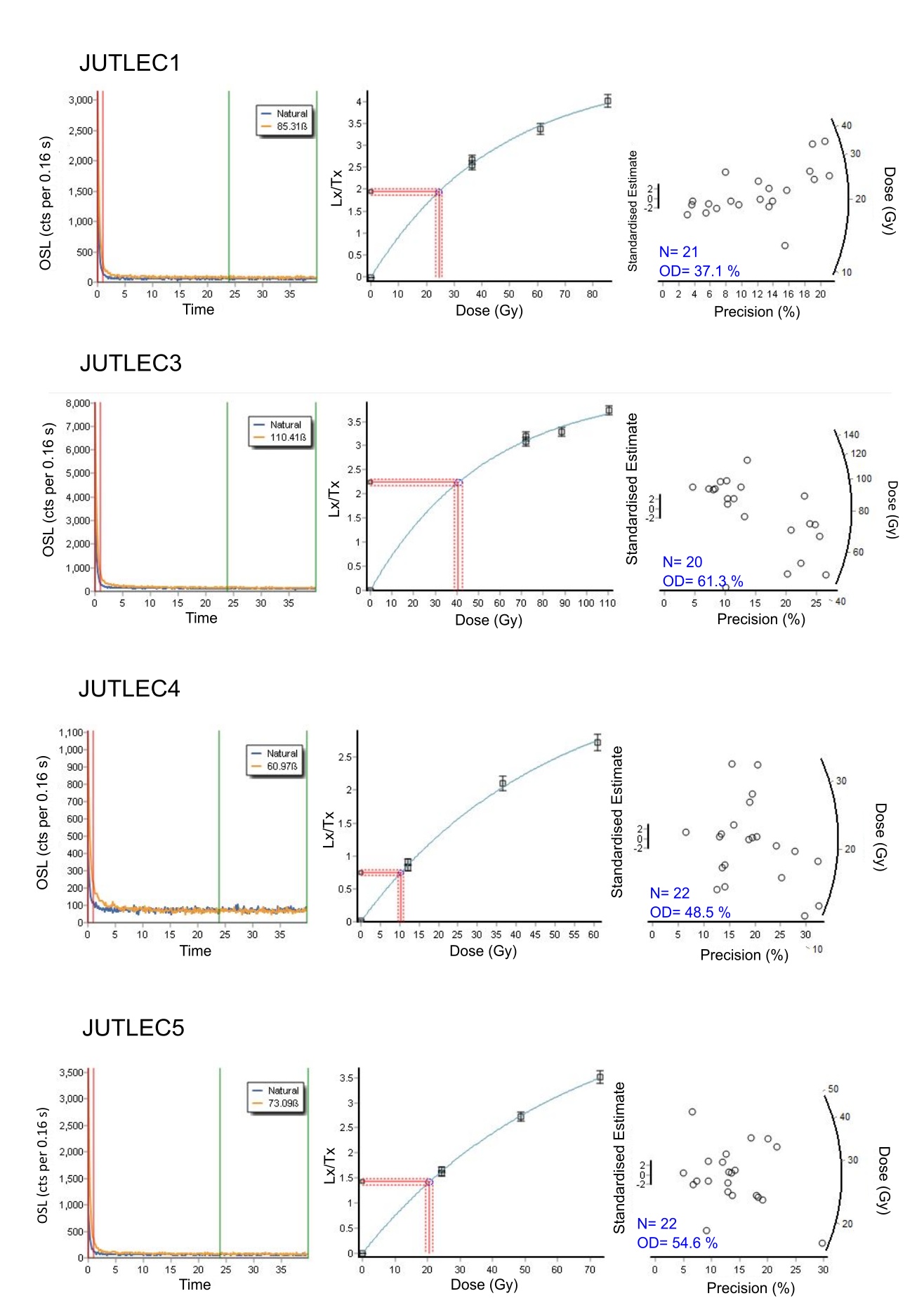


**Figure S10:** Results of the OSL dating showing growth curves, shine down curve and distribution of equivalent dose (De) in histogram from sample number JUTLEC1 and JUTLEC3-5. N – represents the number of aliquots passed the test and OD- regards the over dispersion.


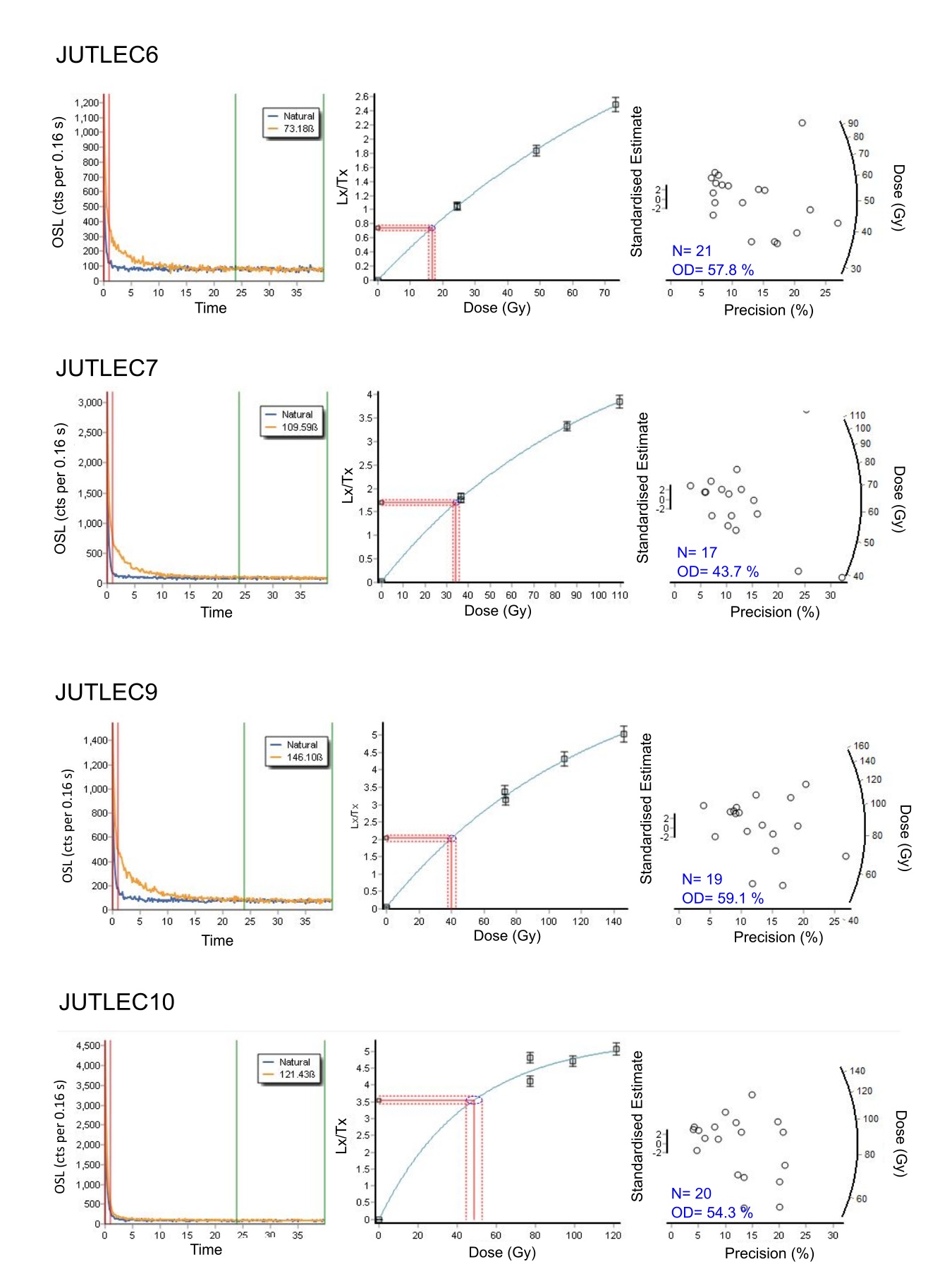


**Figure S11:** Results of the OSL dating showing growth curves, shine down curve and distribution of equivalent dose (De) in radial plot from sample number JUTLEC6-7 and JUTLEC9-10. N – represents the number of aliquots passed the test and OD- regards the over dispersion.


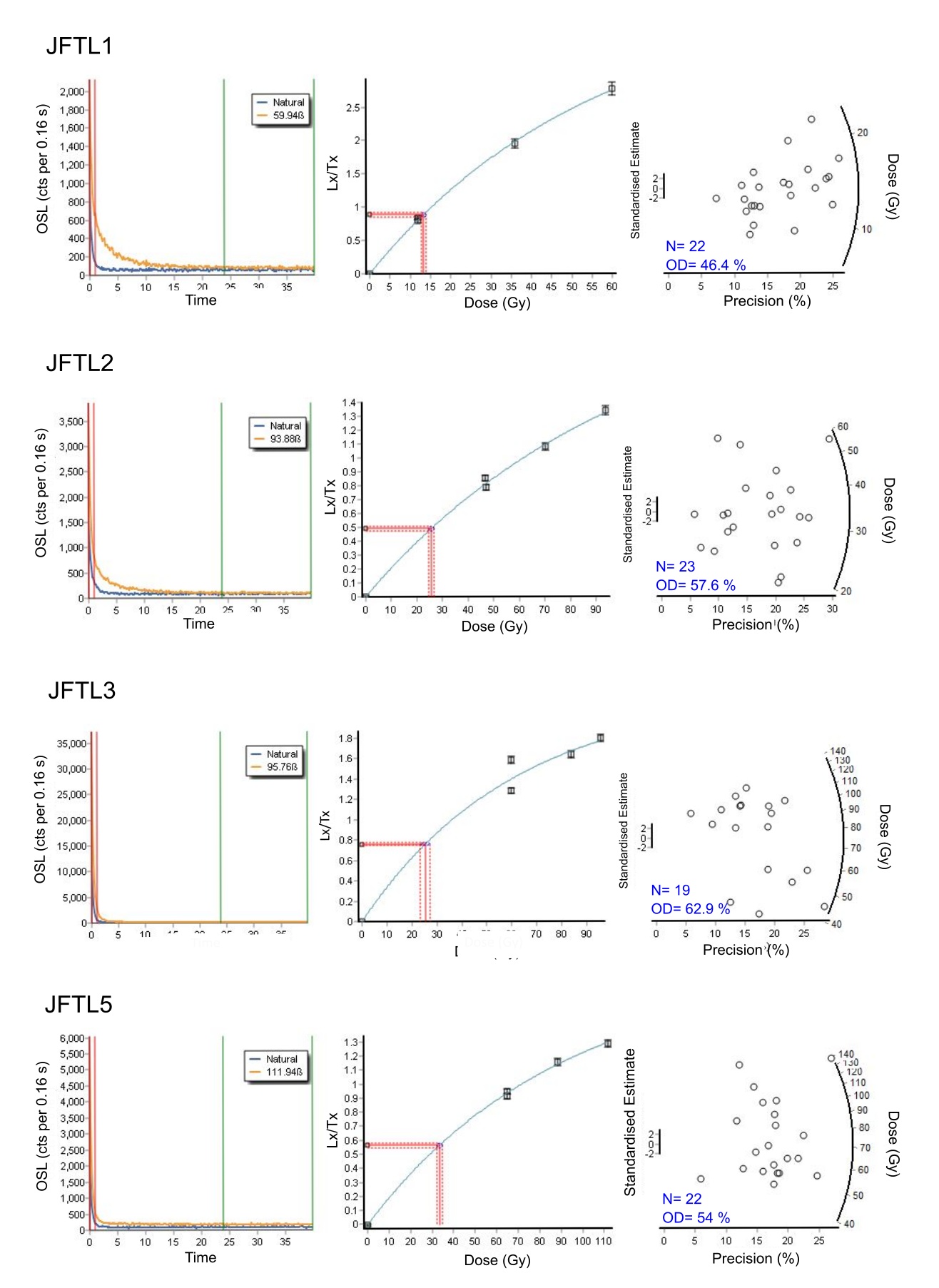


**Figure S12:** Results of the OSL dating showing growth curves, shine down curve and distribution of equivalent dose (De) in radial plot from sample number JFTL 1-3 and 5. N – represents the number of aliquots passed the test and OD- regards the over dispersion.


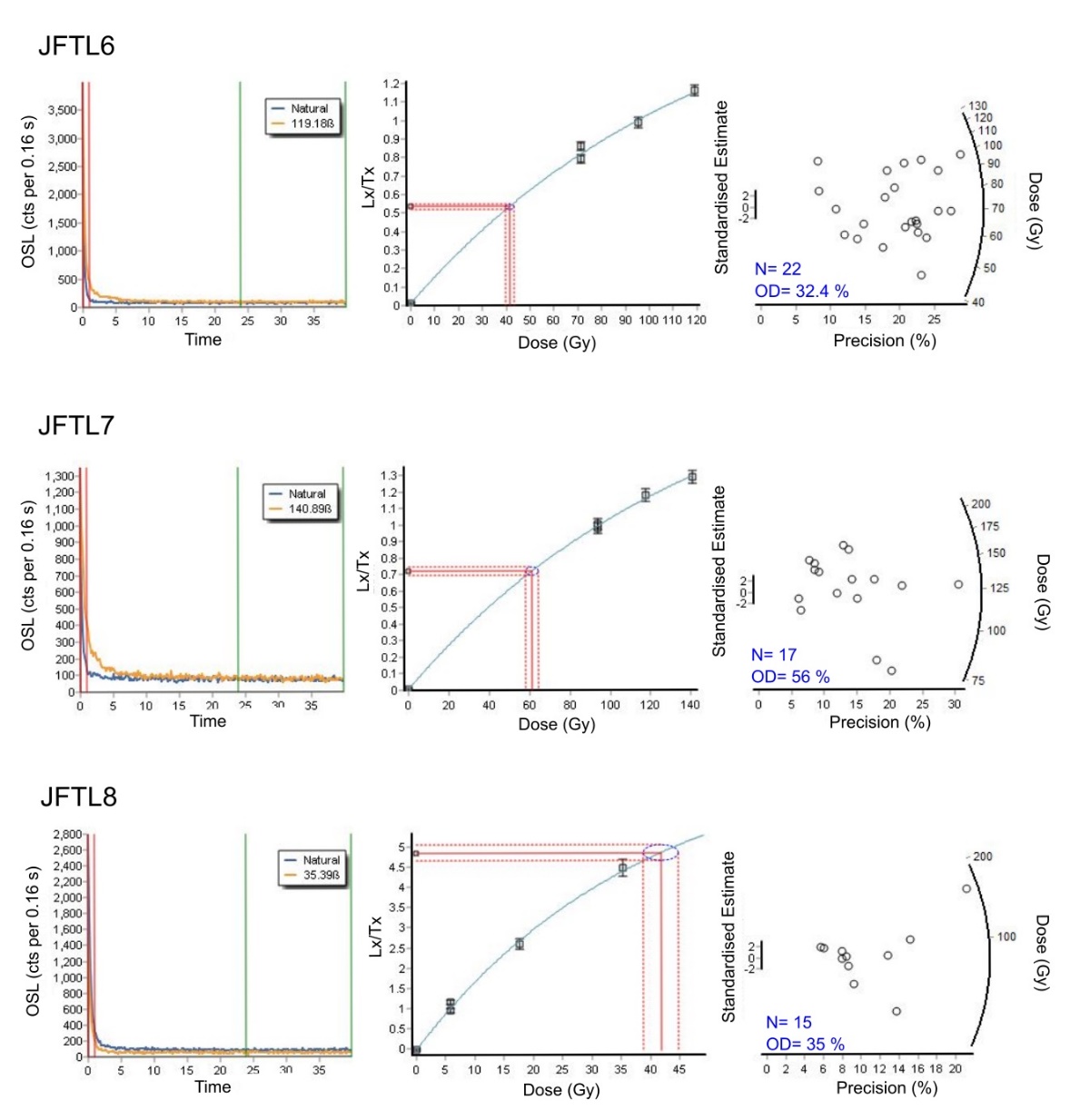


**Figure S13:** Results of the OSL dating showing growth curves, shine down curve and distribution of equivalent dose (De) in radial plots from sample number JFTL6-8. N – represents the number of aliquots passed the test and OD- regards the over dispersion.

**6: References**

1. Malik, J. N., Sahoo, A. K. & Shah, A. A. Ground-penetrating radar investigation along Pinjore Garden Fault: implication toward identification of shallow subsurface deformation along active fault, NW Himalaya. *Curr. Sci.* 1422–1427 (2007).

2. Shaikh, M. A. *et al.* Building tectonic framework of a blind active fault zone using field and ground-penetrating radar data. *J. Struct. Geol.* **155**, 104526 (2022).

3. Pauselli, C. *et al.* Ground penetrating radar investigations to study active faults in the Norcia Basin (central Italy). *J. Appl. Geophys.* **72**, 39–45 (2010).

4. Neal, A. Ground-penetrating radar and its use in sedimentology: principles, problems and progress. *Earth-Sci. Rev.* **66**, 261–330 (2004).

5. Allmendinger, R. W. Inverse and forward numerical modeling of trishear fault-propagation folds. *Tectonics* **17**, 640–656 (1998).

6. Zehnder, A. T. & Allmendinger, R. W. Velocity field for the trishear model. *J. Struct. Geol.* **22**, 1009–1014 (2000).
